# Assessing mortality registration in Kerala: the MARANAM study

**DOI:** 10.1186/s41118-021-00149-z

**Published:** 2022-01-10

**Authors:** Aashish Gupta, Sneha Sarah Mani

**Affiliations:** 1 Harvard Center for Population and Development Studies, Harvard University; r.i.c.e., a Research Institute for Compassionate Economics, 9 Bow Street, Cambridge, MA 02138 USA; 2 Population Studies Center, University of Pennsylvania, Philadelphia, USA

## Abstract

Complete or improving civil registration systems in sub-national areas in low- and middle-income countries provide several opportunities to better understand population health and its determinants. In this article, we provide an assessment of vital statistics in Kerala, India. Kerala is home to more than 33 million people and is a comparatively low-mortality context. We use individual-level vital registration data on more than 2.8 million deaths between 2006 and 2017 from the Kerala MARANAM (Mortality and Registration Assessment and Monitoring) Study. Comparing age-specific mortality rates from the Civil Registration System (CRS) to those from the Sample Registration System (SRS), we do not find evidence that the CRS underestimates mortality. Instead, CRS rates are smoother across ages and less variable across periods. In particular, the CRS records higher death rates than the SRS for ages, where mortality is usually low and for women. Using these data, we provide the first set of annual sex-specific life tables for any state in India. We find that life expectancy at birth was 77.9 years for women in 2017 and 71.4 years for men. Although Kerala is unique in many ways, our findings strengthen the case for more careful attention to mortality records within low- and middle-income countries, and for their better dissemination by government agencies.

**Supplementary Information:**

The online version contains supplementary material available at 10.1186/s41118-021-00149-z.

## Introduction

Records of births and deaths are among the most important statistics that states and societies collect and maintain (Szreter and Breckenridge, 2012). Reliable mortality registration systems help individuals access social and governmental resources, provide timely information to monitor health, and generate essential data to evaluate the determinants of mortality (AbouZahr et al., 2015). These records are the basis of life table databases for high-income countries, such as the Human Mortality Database (Barbieri et al., 2015). Mortality registration, however, is incomplete in many low- and middle-income countries (AbouZahr et al., 2012). These systems miss many vital events, biasing mortality estimates downward (Mathers et al., 2005).

The unreliability and incompleteness of mortality registration in developing countries has motivated demographers to develop and use indirect or survey-based approaches for estimating mortality levels (Department of International Economic and Social Affairs, 1983; Hill, 1991; Moultrie et al., 2013; Timaeus, 1991). These approaches include estimating mortality rates from (a) survival of relatives—often siblings or parents (Brass 1975; Feehan and Borges, 2019; Masquelier, 2013; Masquelier et al., 2020; Saikia et al., 2019; Timæus & Jasseh, 2004); (b) survival in a network, such as by Feehan et al., 2017; (c) inferring mortality from observed age patterns between censuses (Hill et al., 2009); or (d) from model age patterns of mortality (Clark, 2019; Murray et al., 2003; Wilmoth et al., 2012). Many countries have also initiated representative and continuous sample registration exercises (Amouzou et al., 2020; Bhat, 2002; Liu et al., 2016; Setel et al., 2005). One such exercise, the Sample Registration System, has been functional in India since the late 1960s, and annual reports have been published since 1971 (Bhat et al., 1984; Mahapatra, 2017).

Recent evidence suggests that vital registration has also been improving in developing countries (Garenne et al., 2016; Kumar et al., 2019; Rao and Gupta, 2020). India’s Registrar General estimated that about 86 percent of all deaths in the country were registered in 2018 (Gupta, 2020; Banaji and Gupta, 2021). Based on this comparison with the crude death rates in the SRS, ORGI (2020) notes that civil registration was complete in 15 out of 36 states and union territories in India.

Mortality records from areas where civil registration is complete provide several opportunities to improve the evidence base for population health. First, examining how these regions improved registration can provide motivation and strategies for similar regions to improve their vital registration systems. Second, these studies may identify key areas where vital statistics systems can still be improved within the region under study. Third, if mortality records are reliable, and if cause-of-death data are available, these data can serve as the default data for public health surveillance, guiding health policy. Fourth, they provide an impetus for similar evaluations in sub-national areas where mortality registration is complete. Finally, these records may even displace intermittent surveys and indirect estimates and, at the very least, help understand limitations and strengths of alternative demographic estimation techniques.

Building on this perspective, this paper examines mortality estimates from the Civil Registration System (CRS) in Kerala, India. We obtained anonymized records for all deaths registered in Kerala for the years 2006–2017. The death records we obtained contain information on more than 2.8 million deaths. We use estimated population exposures from the DHS Program (2020) for Kerala for this period to estimate age-specific mortality rates. We are able to make three key contributions using these data.

First, we examine the reliability and completeness of death registration in Kerala. We focus on comparisons of overall crude death rates between the Civil Registration System (CRS) and the Sample Registration System (SRS); the extent of missing information on age and sex among registered deaths in the CRS; age misreporting among deaths; comparing female and male mortality rates; and comparisons over time. Our findings suggest improvements in the CRS between 2006 and 2017.

Second, we calculate age-specific mortality rates for the years 2006–2017 using mortality records from the CRS and population estimates from the 2011 Indian census and population projections. Previous studies from India’s CRS have been constrained, because annual reports from the CRS publish death counts by broad age groups (0–1, 1–4, 5–14, 15–24, 25–34, 35–44, 55–64, 65–79, 80+). This necessitates approximations for death counts in abridged life table ages, as done by Rao & Gupta (2020). Access to microdata allows us to compare civil registration system age-specific mortality rates in abridged life table ages to those observed by the SRS. This comparison helps us understand if mortality registration varies by age and sex, and the extent to which concerns about the reliability of the SRS are true in Kerala (Bhat, 2002; Rajan & Mohanachandran, 1999; Saikia et al., 2010). We find that mortality rates generated using the civil registration system show less year-to-year variation than the SRS and are smoother across the adult ages.

Third, we also compare unadjusted mortality rates in single age years computed using census population estimates for 2011 with Gompertz mortality rates in ages 40 to 90. We find very close correspondence between unadjusted and Gompertz mortality rates.

Finally, using these data, we provide the first set of directly estimated annual abridged life tables, to our knowledge, for any state in India. We compile these life tables for 2011 to 2017, the years for which we are able to conduct tests on the robustness of the data.

In the next section, we provide background information on Kerala and the CRS. In ”Materials and methods” section, we discuss the data and estimation methods in detail. We provide our main results and robustness checks thereafter. We conclude with a discussion of the main strengths and weaknesses of these data, suggestions for their better dissemination and further improvement, and, finally, their implications for demographic estimation in low- and middle-income countries.

## Background

A state in south India, Kerala is home to more than 33 million people (Government of India, 2011). Although it is a state in India, Kerala’s population is surpassed by only 42 countries in the world. Its population is also greater than that of 30 countries and areas for which the Human Mortality Database (HMD) makes available life tables (out of a total of 41 HMD countries and areas) (Barbieri et al., 2015; United Nations, 2019b). Kerala’s human development achievements have been widely noted and celebrated (Sen, 2001). It has been an important example within the “routes to low mortality in developing countries” framework (Balabanova et al., 2013; Caldwell, 1986; Kuhn, 2010) and is the state with the highest life expectancy in India (Office of the Registrar General and Census Commissioner of India, 2018). Given these achievements, Kerala’s experience has been important in governance, health, and development policy discussions in India and elsewhere (Heller, 2006; Ramachandran, 1997).

These human development and governance achievements are reflected in Kerala’s CRS as well. Annual reports based on the civil registration system (Office of the Registrar General and Census Commissioner of India, 2013) classify civil registration in Kerala as “complete.”^1^ Recently released summary findings from the fifth round of the National Family Health Survey reinforce these results.^2^ Noting these “high levels of registration” in Kerala, James et al., (2013) argued that “this calls for detailed records of civil registration.”

Within the Government of Kerala administration, the Department of Economics and Statistics and the Department of Local Self-Government maintain the CRS in the state. Civil registration is governed by the Registration of Births and Deaths Act, 1969 and the Kerala Registration of Births and Deaths Rules 1999. The 1999 rules were updated after an initial set of rules in 1970. The departments maintain an IT system to record births, deaths, and marriages, called *Sevana*. The Information Kerala Mission developed this computerized data entry system for civil registration, which started registering births and deaths in 2000 (Government of Kerala, 2008). In the early 2000s, local registration authorities shifted completely to the online system. The Information Kerala Mission organized efforts to enter data from vital records that were not computer recorded. By 2006, all local registrars were using the computerized system for recording vital deaths. In their annual reports, the CRS describes several steps that they take to monitor the quality and completeness of vital statistics. These include spot checks and audits of registrars, checking and following up on data entry errors, and nominal fines for late registration of vital events. A total of 1035 units in the state registered vital events—941 units in rural areas and 94 units in urban areas (Government of Kerala, 2020).

**Fig. 1:**
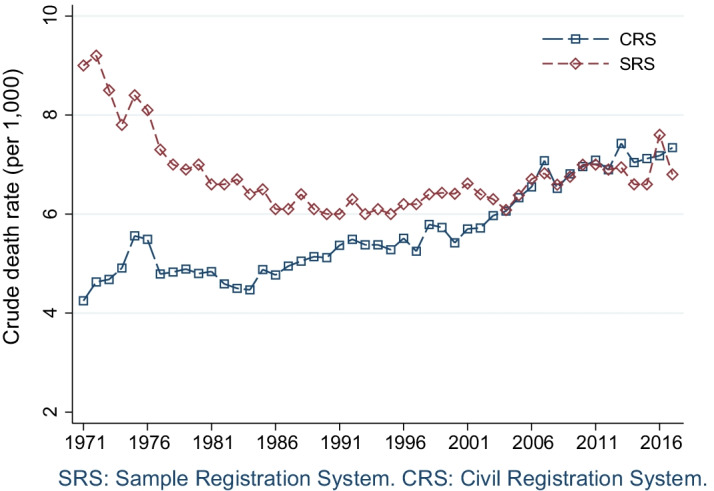
Estimated crude death rates, Kerala, 1971–2017

We present preliminary evidence in Fig. 1 that suggests that the new rules and computerization improved the CRS in Kerala. The figure shows crude death rates from two sources—the CRS and the SRS.^3^ Although higher mortality estimates are not necessarily truer mortality estimates, comparisons from different sources are often used to judge the reliability of mortality reporting (Brown et al., 2019; Preston et al., 2001). Comparisons with the crude death rates observed in the SRS is also the official method used by India’s Registrar General to assess the completeness of death registration (Office of the Registrar General and Census Commissioner of India, 2013). The comparison here for Kerala shows that until the early 2000s, Kerala’s CRS underestimated mortality compared to the SRS. However, starting in the year 2004, and until about 2012, overall mortality rates in Kerala’s CRS were similar to those in the SRS.^4^ More recently, Kerala’s CRS has recorded more deaths than the number of deaths estimated by the SRS. This suggests that the SRS may be under reporting mortality in Kerala and reinforces the suggestion by James et al. (2013) that the CRS may be a useful source to examine mortality levels from individual-level mortality records.^5^

## Materials and methods

The section below describes these mortality data, part of the Kerala Mortality and Registration Assessment and Monitoring (MARANAM) Study, the population exposures we use to calculate mortality rates, and our analytical strategy.

### Data

#### Mortality records

We obtained anonymized individual-level death records from the Department of Economics and Statistics and the Department of Local Self-Government in the Government of Kerala in 2019. Because some records before 2006 are scanned documents which are not machine readable, the Department of Local Self-Government made available death records starting in 2006. The last year for which these records were made available was 2017. These data were anonymized by the Department of Local Self-Government. The data contain information on year of death; age at death; sex of the deceased person; whether the registration authority is a municipal corporation, a municipality, or a *panchayath* (local self-government body in rural areas); the district where the death was registered; and the state where the deceased person’s permanent address is located. We did not obtain any identifying details for any of the deaths that were registered.

This study uses death records and is consequently exempt from review by the Institutional Review Board, because it does not meet the regulatory definition of human subjects research, as noted in 45 CFR Protection of Human Subjects 46.102(e) (in effect on December 22, 2020). According to the U.S. Department of Health and Human Services and Food and Drug Administration regulations, if none of the individuals in a study are alive, it does not involve a human subject and does not require review (US Department of Health and Human Services and others, 2020). We obtained permission to analyze these data and report their results from the Department of Economics and Statistics and the Department of Local Self-Government in the Government of Kerala (dated July 23, 2019). These data are available, on request, directly from these departments.

#### Estimates for population exposures

To calculate life tables and age-specific mortality rates, we use annual counts of population in 5-year age groups estimated by Leddy (2016). These estimates were created by the U.S. Census Bureau for the U.S. PEPFAR program, and are available from the DHS Program Spatial Data Repository (DHS Program, 2020). We adjust these population counts by estimating the 0–1, 1–4, 80–85, and 85+ population to calculate abridged life tables. To do this, we use the estimated population from the 2001 census, the 2011 census, the SRS, and 2016 estimates from India’s official population projections (National Commission on Population, 2019). These sources provide the proportion of 0–5 (or 80+) age group populations within the 0–1 and 1–4 (or the 80–85 and 85+) age groups for the years 2001, 2011, and 2016. For years when these estimates are not available, we use a cubic spline interpolation. Thus, for each year in 2006 to 2017, we obtain estimates of total population by sex in the age groups 0–1, 1–4, 5–10, 10–15, and so on, till 80–85, and 85+.

We check if our results are robust to using alternative sources of population estimates. For this, we compare age-specific mortality rates using the population exposures described above to age-specific mortality rates calculated using the 2011 census estimates and the 2016 population estimates from India’s official population projections, estimated by the National Commission on Population (2019).

#### Other estimates

To assess the reliability of age-specific mortality rates calculated from CRS, we compare these estimates to those from the SRS. The SRS is a continuous survey that has been monitoring vital events in a representative panel of villages and urban blocks since 1971 (RGI, 2016; Mahapatra, 2017). The SRS panel was revised in in 1977–1978, 1983–1985, 1993–1995, 2004, and 2014 (Dreze et al., 2020; ORGI, 2016).^6^ When the SRS panel was revised in 2014, it visited 280 sample units, covering a population of 368,000. In the 2004 revision, the number of sample units was 250, covering a population of 330,000. For comparisons, we created machine readable copies of annual reports from the SRS. We also derive $_{n}a_{x}$ (average person years lived by those who died) values from the 5-year period abridged life tables released by the SRS (Office of the Registrar General and Census Commissioner of India, 2018). For some estimates, such as infant mortality rates and crude death rates for some years for which annual reports were not available online, we used the SRS compendium publication (RGI, 2016). Although the SRS is a survey, standard errors and 95 percent confidence intervals for age-specific mortality rates are not released by the SRS.

In addition to the SRS, we use information from the annual vital statistics reports published by Kerala’s Department of Economics and Statistics (Government of Kerala, 2019). We compute infant mortality rates in ages 0–1 from the fourth round of India’s Demographic and Health Surveys (2015–2016). For robustness checks and comparisons, we also use mortality records from the Human Mortality Database (Barbieri et al., 2015).

### Analytical strategy

Our analytical strategy consists of the following steps. First, we compute crude death rates from the CRS and compare them to the crude death rates from the SRS, examine trends in the proportion of deaths that were missing information on age or sex, and study the severity of age heaping using the Myers blended index and the Whipple index.

In the next step, we compute age-specific death rates from the CRS mortality records and population exposures estimates. We compare these age-specific death rates to those observed by the SRS. Although a variety of methods have been proposed in the literature to infer completeness of civil registration (Hill et al., 2017), we focus on comparisons with the SRS, because this requires fewer assumptions and is more empirical than other methods (Glei et al., 2021).

After this step, we use standard life table methods (Preston et al., 2001) to estimate abridged life tables by sex and calendar-year for the state of Kerala using the CRS age-specific mortality rates.^7^

In robustness tests, we calculate mortality estimates using alternative population exposures; compare infant mortality estimates from the SRS, National Family Health Surveys (NFHS), and CRS; compare unadjusted and Gompertz mortality rates in single-year ages 40 to 90; examine the extent to which migration can bias estimates from the CRS; and, following Glei et al. (2019), compare the relationship between child mortality and old-age mortality for Kerala and the Human Mortality Database countries.

## Results

### Summary statistics for the MARANAM study

Table 1 presents summary statistics for these individual-level mortality data for the years 2006–2017. It shows annual statistics for observed deaths, crude death rates, and the proportion of deaths with missing information on either age or sex. We show observed deaths and estimated crude death rates by year for males, females, and the overall population. The number of deaths observed in Kerala have been increasing, especially in the late 2000s. This could be because of improvements in civil registration but also because of populating aging and past population growth (United Nations, 2019b). Although the SRS crude death rates show greater fluctuations (Fig. 1), increasing crude death rates can be observed in both the CRS and SRS.

**Table 1:** Descriptive statistics: observed deaths, estimated crude death rates, and deaths with missing age or sex

Year	Female		Male		Total		Missing	
	Deaths	CDR	Deaths	CDR	Deaths	CDR	N	Percent
2006	88,131	5.3	125,103	8.0	213,234	6.6	4,333	2.0
2007	97,784	5.8	136,981	8.8	234,765	7.3	3,972	1.7
2008	91,789	5.5	126,037	8.0	217,826	6.7	2,493	1.1
2009	97,334	5.8	132,282	8.4	229,616	7.1	2,272	1.0
2010	101,122	6.0	136,057	8.7	237,179	7.3	2,178	0.9
2011	100,867	6.0	135,283	8.6	236,150	7.2	1,594	0.7
2012	108,298	6.4	127,729	8.1	236,027	7.2	1,305	0.5
2013	110,375	6.5	143,867	9.1	254,242	7.8	1,222	0.5
2014	105,154	6.2	136,376	8.6	241,530	7.4	1,016	0.4
2015	107,509	6.3	136,006	8.6	243,515	7.4	1,166	0.5
2016	111,269	6.5	138,395	8.8	249,664	7.6	1,166	0.5
2017	107,593	6.3	135,461	8.6	243,054	7.4	780	0.3

Calculated from civil registration Data in the Kerala MARANAM Study and population exposures from the DHS Program (2020). CDR is the crude death rate per 1000. Deaths and CDR exclude observations with missing age or sex information. These observations are shown as missing. Four observations were dropped due to missing year

Comparing across gender, female crude deaths rates are lower than male crude death rates in the CRS. This is expected: the SRS also observes vast differences in life expectancy estimates between women and men in Kerala, and these observed death counts could largely be a function of mortality differences rather than reporting differences. Still, the female crude death rate in 2006 was estimated to be 5.3 deaths per 1000, which is lower than other years, suggesting some underreporting of deaths. This could be because this was the first year in which all deaths were supposed to be recorded in the newer computerized CRS.

It is worth noting that the proportion of deaths in which age or sex was missing declined. In 2006, this proportion was 2.0 percent of all deaths registered. By 2017, it had declined to about 0.3of all deaths. This reflects improvements in mortality recording. We provide more detailed patterns on missing information in Additional file 1: Table A1.

### Comparison of age-specific mortality rates in the civil and sample registration systems

**Fig. 2:**
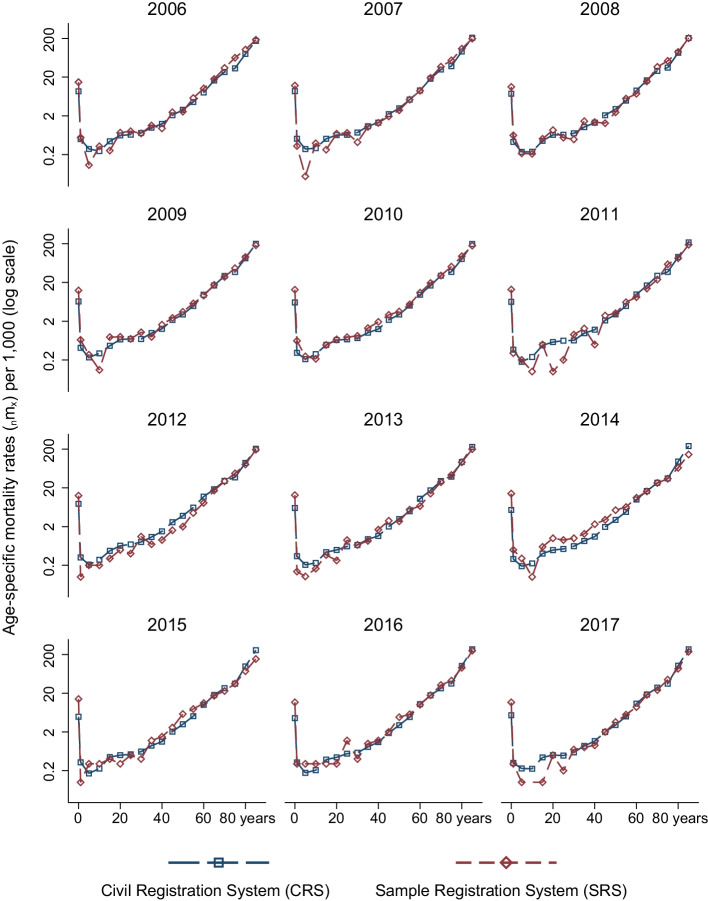
Comparison of age-specific mortality rates ($_{n}$m$_{x}$), female, 2006–2017

**Fig. 3:**
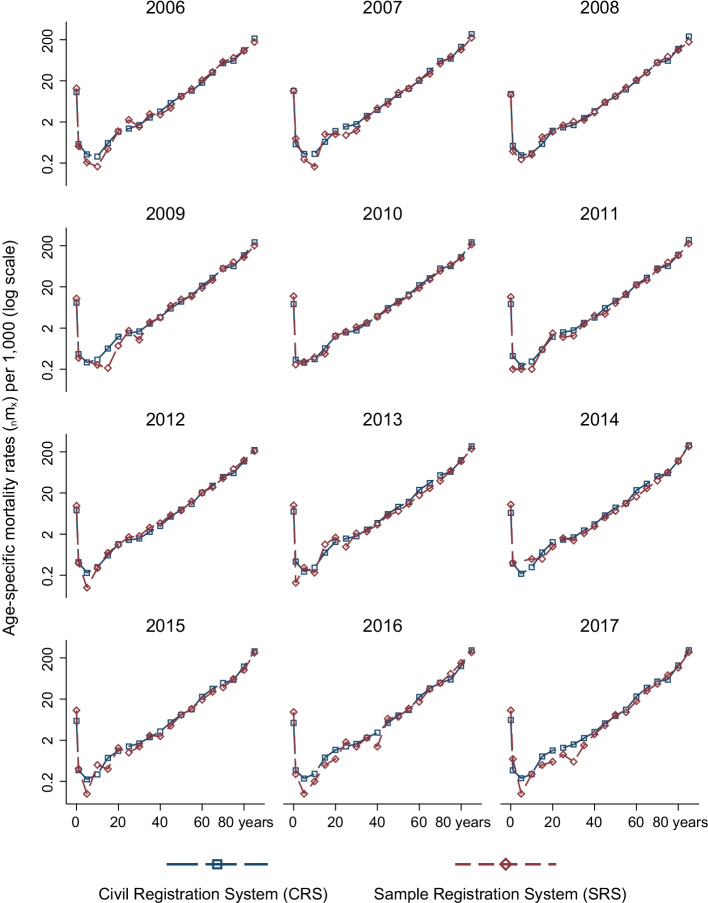
Comparison of age-specific mortality rates ($_{n}$m$_{x}$), male, 2006–2017

We calculate age-specific mortality rates for the years 2006–2017 from the CRS and compare them to those reported by SRS in its annual reports. These comparisons can be seen in Figs. 2 and 3.^8^ The figures, which are on the log scale, show that age-specific mortality rates from the SRS are less smooth than those estimated from the CRS. In contrast to the SRS, the CRS estimates follow expected age patterns of mortality and fluctuate less. A part of the explanation for less smooth estimates in the SRS is that it is a sample. Because the SRS does not publish standard errors of its estimates, we are unable to account for the uncertainty in the SRS estimates. We note that the problems are more severe for women and at younger ages. In some ages, and for some years, age-specific mortality rates in the SRS are very low. A notable exception is the year 2014. The SRS panel was revised in 2014, and mortality rates in the SRS are higher for women than those observed in the CRS. This is not the case for SRS rates for men in 2014.

It is worth noting that CRS estimates reflect expected age patterns of mortality for all years, even in the beginning of the study period. On the other hand, underestimation of mortality in the SRS is increasing within this period. Indeed, SRS estimates fluctuate more in recent years.^9^

In Additional file 1: Figures A5 and A6, we compare 3-year average, 5-year average, and abridged life table (which are for 5-year periods) age-specific mortality rates from the SRS to corresponding CRS rates. Pooling across years in the SRS does reduce fluctuations across ages. However, mortality in some ages, such as between ages 5 and 9 for males, may still be too low in the SRS. In Additional file 1: Table A10, we quantify year-to-year variation in the CRS and SRS age-specific mortality rates. In ages 0–1 and 85+, variation in the CRS and the SRS can be considered similar. For all other ages, variation in the SRS is much higher than in the CRS, likely driven by sampling variability in the SRS.

One age group where mortality rates between the SRS and the CRS differ substantially is below age 1. We examine these differences in greater detail in the ”Robustness” section below, which includes comparisons with the National Family Health Survey-4 in India. More research is needed to understand these differences.

What are the overall implications of the comparison of age-specific mortality rates between the SRS and the CRS? One interpretation is that these findings are contrary to the expectation that civil registration systems in developing countries necessarily perform worse than other mortality monitoring systems, such as survey-based estimates from sample registration systems. These comparisons reveal a need for careful assessment of mortality estimates from the SRS, as well as of associated studies, such as those examining causes of death based on verbal autopsies of deaths recorded by the SRS.

### Life table estimates

Table 2 provides estimates for age-specific probabilities of death between ages 0–15, ages 15–60, and 60–85 by year. Mortality rates have declined in the child and adult ages, especially when comparing estimates from 2007 to 2017. Patterns of older age mortality are more complicated, with no clear trends. This may be driven by age misreporting and variation in registration of deaths. Probability of death for all ages was lower in 2006 compared to 2007, especially in the adult and older ages. This may be because of under-reporting of deaths in 2006.

**Table 2:** Probability of dying between ages 0–15, 15–60, and 60–85; by year and sex

Year	Probability of dying					
	Ages 0–15		Ages 15–60		Ages 60–85	
	Female	Male	Female	Male	Female	Male
2006	0.0132	0.0158	0.0685	0.1679	0.5659	0.7508
2007	0.0134	0.0169	0.0750	0.1825	0.6172	0.8022
2008	0.0114	0.0147	0.0716	0.1723	0.5879	0.7684
2009	0.0107	0.0132	0.0718	0.1755	0.5941	0.7726
2010	0.0097	0.0121	0.0723	0.1808	0.5893	0.7584
2011	0.0098	0.0120	0.0703	0.1794	0.6103	0.7798
2012	0.0114	0.0119	0.0851	0.1575	0.6110	0.7566
2013	0.0096	0.0115	0.0697	0.1783	0.6200	0.7880
2014	0.0086	0.0107	0.0665	0.1688	0.6083	0.7732
2015	0.0082	0.0099	0.0695	0.1636	0.6174	0.7714
2016	0.0077	0.0094	0.0658	0.1582	0.6243	0.7788
2017	0.0089	0.0103	0.0682	0.1580	0.6307	0.7891

Authors’ calculations using data from the Kerala MARANAM Study and adjusted population estimates from the DHS Program (2020)

Table 3 provides life expectancy estimates for the years 2011–2017. We report the full set of life tables for these years in Additional file 1: Tables A2–A8. Consistent with the SRS, female life expectancy at birth is much higher than male life expectancy for 2006 to 2017. These patterns can be seen across the life course. Surprisingly, life expectancy estimates for 2017 are lower than those of other recent years. Additional file 1: Figure A2 also notes an increase in the infant mortality rate for 2017 in the CRS. These patterns may reflect true mortality increases, given the large scale increase in poverty and unemployment in India following the demonetization in India in November 2016 (Dreze et al., 2020).

**Table 3:** Estimates for life expectancy at birth, age 15, age 60; by year and sex

Year	Life expectancy at birth					
	Female	Male	Age 15		Age 60	
			Female	Male	Female	Male
2011	78.2	70.9	64.0	56.7	21.4	17.0
2012	77.5	72.3	63.3	58.1	21.2	17.9
2013	78.0	70.8	63.8	56.6	21.1	16.9
2014	78.4	71.4	64.1	57.1	21.3	17.1
2015	78.2	71.6	63.8	57.3	21.1	17.2
2016	78.2	71.7	63.8	57.4	20.9	17.1
2017	77.9	71.4	63.6	57.2	20.8	16.8

Authors’ calculations using data from the Kerala MARANAM Study and adjusted population estimates from the DHS Program (2020)

## Robustness

### Comparison of infant mortality rates

A pattern that can be noticed in Figs. 2 and 3 is that mortality rates below age 1 in the SRS are higher than those observed in the CRS. To investigate this, we compare mortality rates in the SRS, CRS, and the fourth round of the National Family Health Survey (NFHS) in Additional file 1: Figure A2.^10^ NFHS and CRS rates show declines over time, whereas SRS age-specific mortality rates fluctuate much more and increase substantially between 2008 and 2015. It is likely that these fluctuations would be less severe if the SRS were to visit a larger number of clusters.

In addition, NFHS mortality rates for infant girls are very close to those in the CRS, while rates for infant boys in the NFHS are between the CRS and the SRS. The SRS shows lower mortality among boys than among girls. In contrast, mortality among boys compared to girls is higher in the NFHS and the CRS. This is confirmed by Figure A3. Infant mortality rates for females are observed to be lower than those of boys across contexts (Alkema et al., 2014; Sawyer, 2012). Although this is not true across India, Kerala is a notable outlier in the Indian context (Sen, 2001), with females enjoying higher status compared to other states. In light of this, patterns in the NFHS and CRS are more likely to be true than those in the SRS. Recently released NFHS-5 state fact sheets do have information on sex-specific probability of dying by age 1. Still, they note that overall infant mortality ($_{1}$q$_{0}$) in the period 2014–2019 to be 4.4 deaths per 1000 births (Varghese et al., 2021). This is the same level of mortality below age 1 in the CRS and is much lower than that of the SRS.

We note that there are some unexplained patterns in the CRS as well. There is an unexplained rise in mortality among girls in 2012, which is not seen for boys. Reasons for this are not clear. According to the CRS, infant mortality also increased slightly in 2017 compared to 2016. This, however, is similar to patterns found elsewhere by Dreze et al. (2020) and may be due to the income and employment shocks induced by India’s demonetization experiment.^11^ When further data is available, such as from NFHS-5, it would be helpful to understand infant mortality in Kerala.

### Alternative population exposures

To what extent are these estimates driven by problems with underlying exposures, rather than mortality? Additional file 1: Figures A7 and A8 examine the implications of alternative population exposures. In Additional file 1: Figure A7, census-based age-specific mortality rates are calculated using deaths in the period from September 1, 2010 to August 31, 2011 in the numerator and Census 2011 population as on March 1, 2011 in the denominator. In Additional file 1: Figure A8, we use the 2016 population as estimated by National Commission on Population (2019) for comparison. Both figures reveal that using alternative population exposures changes estimated mortality rates slightly, but does not alter any of the substantive conclusions drawn here.

### Age heaping

Additional file 1: Figures A9, A10 describe the age distribution of deaths in single age years for females and males, respectively. These figures show a high-degree of age heaping, even in a universal literacy context. However, age heaping has been declining over time, as can be observed in less sharp peaks in later years.

To systematically assess the extent of age heaping and digit preference, we report the Myers blended index and Whipple index. The Myers index considers preference of ages ending in each of the digits 0 to 9. The index ranges from 0 to 90, with 0 corresponding to no age heaping and 90 corresponding to extreme age heaping. Whipple’s age heaping index indicates the preference of ages ending in 0 or 5 for ages between 23 and 62. The index ranges from 100 to 500, with 100 indicating no digit preference in age reporting, while 500 corresponds to all reported ages ending in digits 0 or 5. Figure A11 reports these indices. We observe a downward trend for the indices between 2006 and 2017, indicating that over time, there is a fall in digit preference in ages reported during death registration. The digit preference in reported ages is higher for females than males, but the gap has reduced over time. For recent years, age heaping in deaths registered in Kerala is less severe than the age heaping reported in the Indian census in 2011 (United Nations, 2019a).^12^

### Comparison with Gompertz mortality rates

Given age misreporting in both population and death counts, to what extent are the life expectancy estimates at older ages biased? Although they are affected by age misreporting, Figs. 2 and 3 show that broadly, mortality in the abridged life table ages increases exponentially in the adult ages for the mortality rates estimated using the CRS. However, using abridged life table ages masks considerable age heaping, as reported in the section above. To understand the extent to which age misreporting in single ages affects life expectancy estimates, we compute Gompertz mortality rates using single age population counts from the 2011 census and deaths in single ages from the CRS data. The results of this comparison are shown in Additional file 1: Figure A12.

Each panel in Additional file 1: Figure A12 shows estimates for females and males. The top-left panel shows mortality rates, the top-right panel shows survivors to age x, the bottom-left panel shows life table deaths, and the last panel shows estimated life expectancy. Together, the panels show that even with substantial age heaping and misreporting of ages, unadjusted life expectancy estimates are very close to life expectancy estimates from predicted mortality rates using a Gompertz function. In practice, our mortality rates are less affected by age misreporting than the ones reported here, because we use 5-year age intervals. Even then, the similarity of unadjusted and Gompertz life expectancy estimates in single ages and up to age 90 is reassuring.^13^

### Comparison of the relationship between child and older age mortality

Following Glei et al. (2019), Additional file 1: Figure A14 plots the relationship between child mortality $_{5}q_{0}$ and older age mortality $_{20}q_{60}$ for Kerala (2006 to 2017) and all populations available in the Human Mortality Database (HMD) for the period 1971 to 2017. Among older adults, the HMD estimates are clustered along a line connecting the upper right (high child and old age mortality) and lower left quadrants (low child and old age mortality). Given $_{5}q_{0}$, $_{20}q_{60}$ estimates for Kerala are at levels similar to those of the HMD countries and areas. These comparisons suggest that the possibility of biases in levels of $_{5}q_{0}$ or $_{20}q_{60}$ in Kerala is relatively low. Additional file 1: Figure A13 shows these patterns for adult and child mortality. It also finds that patterns observed in Kerala are not anomalous when compared to the HMD.

### Migration

We include a discussion of how migration may affect the mortality rates in Additional file 1. Briefly, because Kerala is a high-migration context (Rajan and Zachariah 2019), it is important to consider it in a mortality analysis. However, data constraints do not permit a full accounting of how mortality among in- and out-migrants affects estimates here. In the Statistical Appendix, we show that deaths among out-migrants outside Kerala are likely a minuscule fraction of overall deaths. We also use existing data to investigate how the crude death rate will change if migration is accounted for. We find these changes to be small. We note that a careful accounting of how migration affects mortality rates would also warrant an assessment of usual residence status in Kerala for both deaths and population. This information is not available right now.

## Discussion

Using vital statistics death records, this article provides the first set of annual age-specific mortality rates and abridged life tables by sex for any state in India. The article extends the existing literature on civil registration in India (Gupta et al., 2016; Rao and Gupta 2020), which has been constrained by the unavailability of death counts in abridged life table age groups in CRS summary reports. We document a large sex difference in mortality, with much lower life expectancy for men than for women, which is important to understand from a public health perspective. More broadly, the article shows that just as there are routes to low mortality in developing country contexts (Caldwell, 1986; Kuhn, 2010), reliable and complete civil registration systems within developing countries do exist. In addition to mortality estimation, these systems are critical from a human rights perspective, because they help people access their basic rights (Bhatia et al., 2019; Szreter and Breckenridge, 2012).

The article demonstrates that the CRS in Kerala records more deaths than those estimated by the SRS. This raises concerns about the current functioning of the SRS. Age-specific mortality rates from the CRS are smoother for annual periods than those estimated by the SRS. This suggests that an increase in the overall sample size of the SRS, as well as an increase in the number of clusters it visits, is necessary for reliable annual estimates. We note a discrepancy in mortality rates below age 1 between the SRS, the CRS, and the NFHS, which needs further investigation. More transparency from the SRS and public release of its microdata are essential to understanding these discrepancies.

In robustness checks, we show that mortality estimates from the CRS are robust to alternative estimates for populations exposed to the risk of death, that accounting for migration will not change the results reported here substantially, and that age heaping and misreporting have been declining in the CRS in Kerala. Age heaping may still be influencing mortality estimates (Gerland, 2014), particularly at older ages (Preston et al., 1999). In this article, we have preferred empirical estimates over smoothing techniques, largely because simpler and intuitive estimation techniques are more likely to be adopted by civil registration authorities. These authorities often lack technical expertise or resources for more complicated analyses on a routine basis. Still, we show that smoothed mortality rates using a Gompertz model predict similar levels of mortality as unadjusted rates. Overall, the analysis here reinforces conclusions drawn in Rao and Gupta (2020): that the CRS can be used to monitor trends and determinants of mortality reliably in some contexts within India.

We note that these data and our analyses have some limitations. For some of the earliest years for which this data is available, mortality probabilities are slightly lower compared to later years. Given that there are no identified reasons to expect an increase in mortality in this period, particularly between 2006 and 2007, estimates for earlier years likely underestimate mortality. There are also a higher number of deaths with missing information on age and sex in the years 2006–2010. This suggests more careful use of data for these years. We recommend that data from these earlier years (such as before 2011) should largely be ignored in mortality analyses, particularly for analysis of trends. These data may still be valuable for understanding determinants of health, but conclusions drawn from these years may be tentative. Other discrepancies, such as an increase in infant mortality among girls in 2012, also deserve more scrutiny.

From the perspective of improving the CRS in Kerala, the article identifies several priorities. Within Kerala, these data and their properties deserve greater attention than they currently receive. Although government agencies prepare an annual report based on these data, they are not used to estimate life expectancies or understand the determinants of mortality.^14^ In this, there is a case for improving capacity within government agencies to make fuller use of these data (Bhatia et al., 2021).

During the ongoing COVID-19 pandemic, civil registration data from Kerala did not find excess mortality in 2020 (Government of Kerala, 2021). This is partly because the scale of additional mortality in Kerala was not that high in 2020 and partly because of delays in registration of deaths (Gupta et al., 2021). For 2021, however, Kerala’s CRS did record more deaths than expected: excess mortality was even greater than the reported COVID-19 death toll in the state (Kumar, 2021; Rajendran and Kurian, 2021; Ramani, 2021). These data have informed both local and comparative perspectives on COVID-19 mortality. The magnitude of excess mortality in Kerala is lower than other Indian states where such data is available, likely reflecting better management of the pandemic within the state (Banaji and Gupta, 2021). They have also bolstered calls within the state to better monitor and address the mortality burden. Moving forward, if these data are released, they can inform scientific and policy questions about mortality impact, spread, and age gradient.

In terms of reforms within the system, annual reports from vital registration systems at both the state and the national levels currently summarize data on deaths in broad age categories (0–1, 1–4, 5–14, 15–24, 25–34, 35–44, 45–54, 55–64, 65–79, and 70+). Ideally, annual reports should consider displaying death counts and mortality rates by abridged life table ages, going up to age 85. These reports are also compiled after much delay.^15^ Increased capacity in compiling these reports and releasing them sooner will also help serve the goals of public health surveillance. The analyses presented here build a case for further use of these data to understand the distribution and determinants of health in Kerala. The CRS in Kerala may also allow for finer estimates of mortality, such as at the district level; understanding the quality of cause-of-death reporting, and, if reliable, comparisons based on verbal autopsy based studies; and understanding social patterns, such as by gender and religion. Unlike other Indian administrative systems, the CRS does not currently collect information on caste and Indigenous status. Given that these are important determinants of population health in India (Gupta and Sudharsanan, 2020; Ramaiah, 2015; Vyas et al., 2021), reforms in civil registration should prioritize collecting this information.

The analyses here also have implications for research on civil registration and mortality estimation in developing countries in general, as well as implications specific to Kerala and the Indian context. The analysis here supports efforts to reduce reliance on modeled estimates, which have considerable uncertainty (Rao et al., 2021). Kerala’s experience in improving Civil Registration System may be helpful for other states in India in improving their civil registration systems. Our results make a case for much greater engagement with vital statistics systems in low- and middle-income country contexts.

## Supplementary information

**Additional file 1.** Supplementary Material.

## Data Availability

Individual level mortality data are directly available from Department of Economics and Statistics, Government of Kerala. The authors did not receive permission to share these data publicly. Population exposures and other data are publicly available, through the websites of the Indian Census, the Human Mortality Database, and the Demographic and Health Surveys. Replication files not containing original data, but which replicate the results, will be posted publicly.

## Footnotes

1 This comparison is based on comparing crude death rates from the Civil Registration System (CRS) with those from the SRS (SRS).

2 National Family Health Survey (NFHS)-5, in 2019–2020, asked a question on the deaths in the household in the last 3 years. For previous rounds, this question has been found to estimate reliable mortality rates at the national level (Gupta and Sudharsanan, 2020). In addition to the household death question, NFHS-5 asked about the proportion of these deaths that were “registered with the civil authority.” This proportion was 97.4 percent (Government of India, 2020).

3 SRS crude death rates rates are collated from SRS annual reports and compendiums. CRS rates are collated from annual reports of the CRS (Office of the Registrar General and Census Commissioner of India, 2013) and reports published by the Department of Economics and Statistics, Government of Kerala (Government of Kerala, 2009). The CRS rates use official projections from the Indian census for population exposures. It is possible that a part of the difference between the CRS and SRS is due to differences in underlying population exposures. Unfortunately, the population estimates and survey weights which the SRS uses for calculation of crude and age-specific mortality rates are not available in the public domain, and thus direct comparison using the same population estimates cannot be made.

4 We note that this study identifies the early 2000s as an important period in which mortality registration improved dramatically, but causes for this improvement are not exactly clear. The computerization of vital registration in Kerala in this period certainly would have helped. It is possible that earlier estimates of crude death rates from the CRS are underestimates, because some death certificates were issued as paper certificates by local officials but were not recorded in centralized databases that were reported to the Office of the Registrar General and Census Commissioner of India. Computerization of registration would reduce the extent to which these paper death certificates are missed. An additional factor could be state initiatives. According to the 2009 all-India CRS Report, Kerala was one of only three states that had modest fines for delayed and non-registration of deaths (Office of the Registrar General and Census Commissioner of India, 2013). Other initiatives, such as information campaigns and audits of registration authorities in Kerala, are discussed in multiple CRS reports. However, further qualitative research and interviews would be useful in understanding the relative contribution of these and other factors in improving the CRS in Kerala.

5 Another source of difference can be how the different sources treat deaths of migrants. We examine the extent to which migration may affect CRS rates in Additional file 1: Appendix. Our analysis suggests that excluding migrants from the CRS does not change overall crude deaths rate substantially. The SRS estimates mortality rates for usual residents, but the definition of a usual resident is not available from the SRS reports.

6 Information on the extent to which older sampling units were discarded in these revisions is not available. For the revision in the 1980s, Bhat et al. (1984) note that “about 58 per cent of the old sample units based on the 1961 census were replaced by new sample units drawn from the 1981 census frame” (p. 39).

7 We borrow sex-specific $_{n}a_{x}$ values from the SRS abridged life tables for Kerala. The $_{n}a_{x}$ estimates for 2006 come from the 2004–2008 SRS abridged life tables, for 2007 from the 2005–2009 SRS abridged life tables, and so on. We do not redistribute the deaths with missing age or sex information, nor do we use any smoothing techniques. We also calculate the probability of death for the ages 0–15, 15–60, and 60–85.

8 We do not redistribute the deaths that have missing information on age or sex. We drop these observations.

9 This deterioration in the SRS to capture mortality could be because of changes in the SRS panel, or because of difficulty in capturing deaths as mortality declines. If underlying individual level SRS data are available, this could be examined in detail. Additional file 1: Figure A1 reports ratios of mortality rates between the CRS and the SRS by age.

10 The NFHS is India’s version of the Demographic Health Surveys (DHS). We calculate age-specific mortality by year in the NFHS by using the approach outlined in Moultrie et al., (2013) and Gupta (2020b). In particular, we estimated person years lived and deaths in the age group 0–1 for the individual years for 2004–2015.

11 The SRS also does not provide estimates of sex-specific infant mortality rates in its annual reports. In Additional file 1: Figure A4 we compare estimates for infant mortality rates from the SRS with those from the CRS (calculated by dividing infant deaths and births in a year). This figure also confirms that patterns observed in Additional file 1: Figure A2 are not due to biases in the estimation of the 0–1 population.

12 The UN Demographic Yearbook 2018 calculates the Whipple index for the Indian census of 1991, 2001, and 2011. The Whipple index for the Indian census population has improved over the decades from 290.3 in 1991, to 230 in 2001, to 171 in 2011.

13 Given the additional similarity in mortality rates when using Census 2011 population and adjusted population estimates from the DHS Program (2020), as reported in the ”Alternative population exposures” section, it is reasonable to argue that mortality rates and life tables reported for other years will not substantially change if single-year population exposures were available for those years and Gompertz mortality rates were calculated instead of unadjusted mortality rates.

14 Reports from the vital statistics system (Government of Kerala, 2019) do report crude death rates, the infant mortality rate (calculated by dividing infant deaths and births), and other indicators, such as the sex ratio at birth.

15 At the time of writing, April 2021, the last available reports are for 2018.
