# Supplementary material for: Assessing mortality registration in Kerala: the MARANAM study
Source: Genus. 2022 Jan 10;78(1):1. doi: 10.1186/s41118-021-00149-z (PMC8744029; doi:10.1186/s41118-021-00149-z)
Supplement: Supplementary file 1 — Additional file 1. Supplementary Material. [file 41118_2021_149_MOESM1_ESM.pdf]

SUPPLEMENTARY APPENDIX

Migration and mortality estimates

Kerala is a high-migration context. In 2011, the Kerala Migration Survey estimated that 2.28 million emigrants from Kerala lived across the world (Rajan & Zachariah, 2019).[1] It is not clear the extent to which the deaths of migrants from Kerala are missed by the Civil Registration System (CRS) and the degree to which these deaths bias mortality estimates reported here. These deaths should be included if the people dying were usual residents of Kerala. Similarly, the population estimates in the denominator of our mortality calculations are also for usual residents of Kerala.[2]

International migration

Based on data we have access to, we do not know the exact number of people who died outside India but were usual residents of Kerala to include them in our mortality estimates. The closest estimate available is the overall death counts provided by Indian embassies for 2009 to 2016. In 2009, 1,222 deaths were registered at all Indian consulates and missions abroad. This number was 1,652 deaths in 2010, 1,745 deaths in 2011, 1,754 deaths in 2012, 1,106 deaths in 2013, 4,161 deaths in 2014, 4,160 deaths in 2015, and 4,266 deaths in 2016. These total deaths are a small fraction of overall deaths in the CRS. Indeed, the highest number of deaths was in 2016, and these deaths are just 1.7 percent of the total deaths registered in that year. Although it would be useful to understand mortality patterns among migrants from Kerala, it is likely that additional information on these deaths will not alter the estimated mortality rates and life expectancy values.

Internal migration

Kerala is ranked above other Indian states in its performance on migrant-friendly policies and attracts migrant labor from other parts of India (Bhagat & Keshri, 2020). In 2011, the Indian census counted about 683,828 individuals who were living in Kerala but were born outside the state (Office of the Registrar General and Census Commissioner of India, 2011). The census also counted 654,423 individuals whose last residence was outside Kerala but within another state of India. Of these, about 80,045 individuals' last place of residence was outside the state of Kerala in the last year. In Appendix Table A9, we examine changes in the crude death rate for Kerala if these individuals are removed from the denominator. We also examine the extent to which the crude death rate changes if we exclude those deaths for persons whose state of permanent residence was not Kerala according to the CRS. In general, Appendix Table A9 shows that excluding those with permanent residence reported to be outside Kerala, people born outside Kerala, or people whose last residence was outside Kerala in the last year does not change the estimated crude death rate substantially. It is worth noting that some of the people excluded here may in fact be usual residents. If true estimates of usual residents among those who are alive or dead are available, changes in the crude death rates will likely be smaller than the ones reported here.

References

Bhagat, R. B., & Keshri, K. (2020). Internal migration in India. In *Internal migration in the countries of asia* (pp. 207–228). Springer. http://dx.doi.org/10.1007/978-3-030-44010-7_11

DHS Program. (2020). *Spatial Data Repository - Population Estimates.* Retrieved 2020-12-25, from https://spatialdata.dhsprogram.com/population-estimates/

Office of the Registrar General and Census Commissioner of India. (2011). Census of India 2011: Household listing Operations. *Office of the Registrar General Census Commissioner, India. Indian Census Bureau*.

Rajan, S. I., & Zachariah, K. C. (2019). *Emigration and remittances: New evidences from the Kerala migration survey 2018* (Tech. Rep.).

Figure A1 Ratio of mortality rates from the civil and sample registration systems

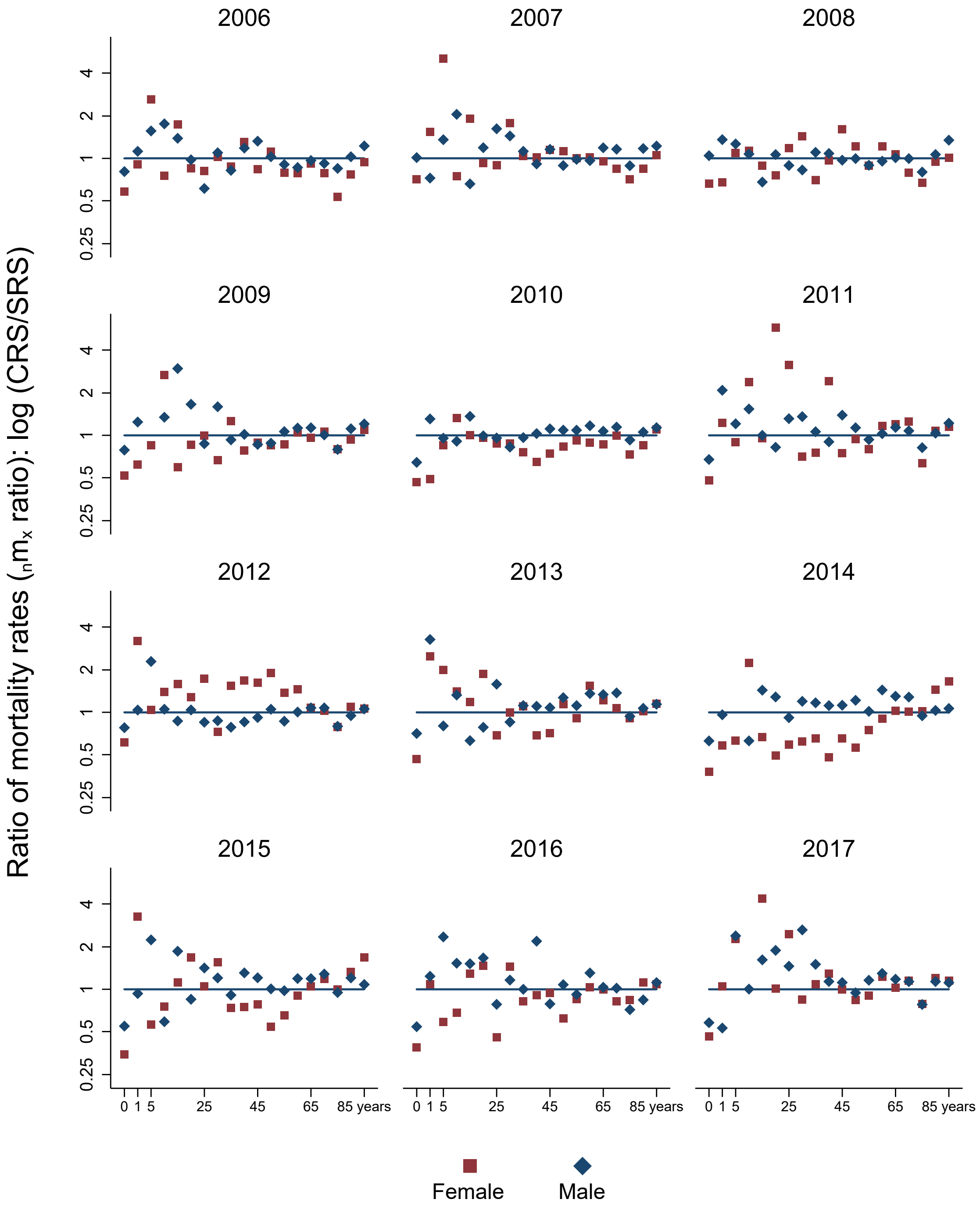

Sample Registration System (SRS) estimates are from SRS annual reports. Civil Registration System (CRS) estimates are calculated from observed CRS deaths and estimated mid-year population.

Figure A2 Comparison of estimates of ${}_1m_0$

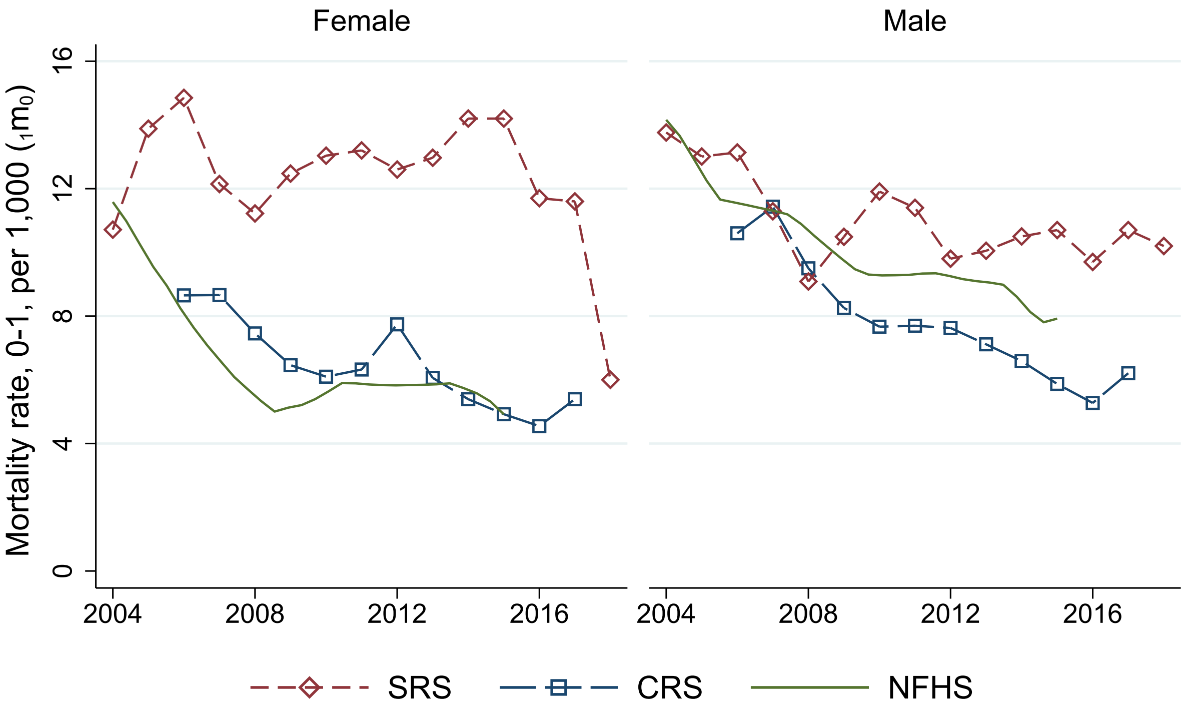

National Family Health Survey (NFHS) estimates are smoothed and reflect two-year moving averages. NFHS estimates are calculated by estimating person years lived and deaths in each year. Sample Registration System (SRS) estimates are from SRS annual reports. Civil Registration System (CRS) estimates are calculated from observed CRS deaths and estimated mid-year population.

Figure A3 Comparison of sex ratios of ${}_1m_0$ (boys v girls)

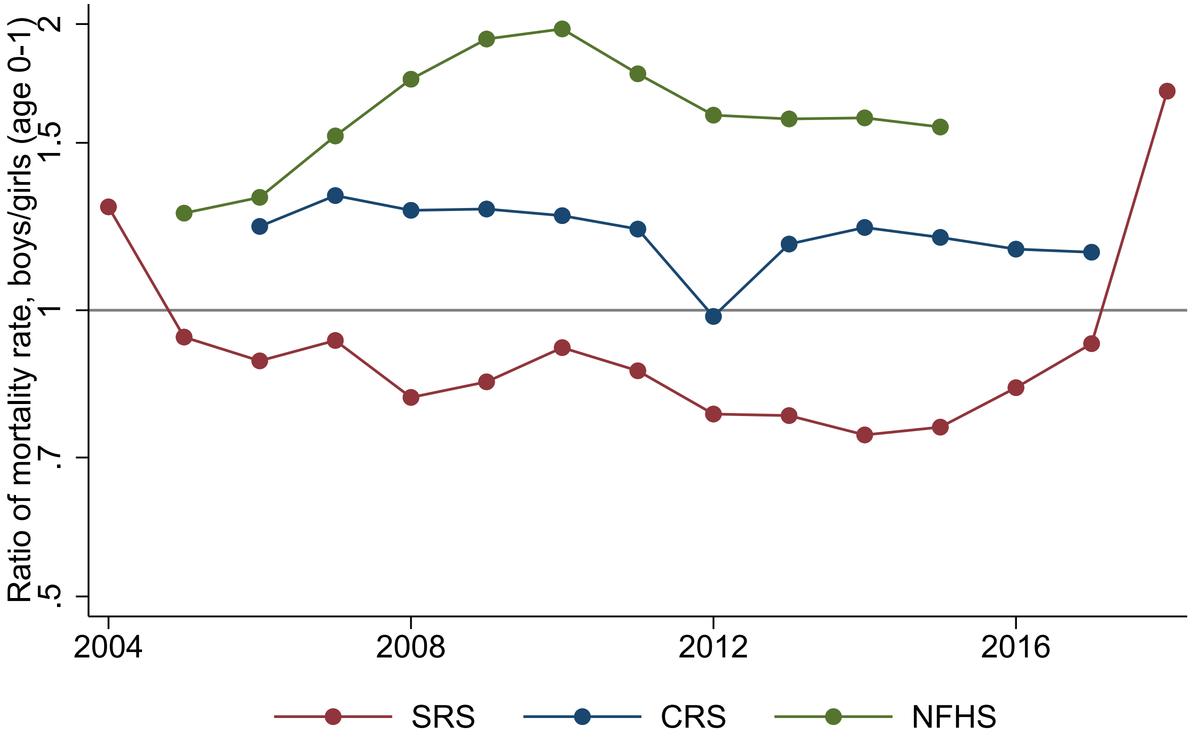

Ratio shown is mortality rate for boys divided by mortality rate for girls in ages 0-1. National Family Health Survey (NFHS) estimates are smoothed and reflect two-year moving averages. NFHS estimates are calculated by estimating person years lived and deaths in each year. Sample Registration System (SRS) estimates are from SRS annual reports. Civil Registration System (CRS) estimates are calculated from observed CRSdeaths and estimated mid-year population.

Figure A4 Comparison of estimates of infant mortality rate from civil and sample registration systems

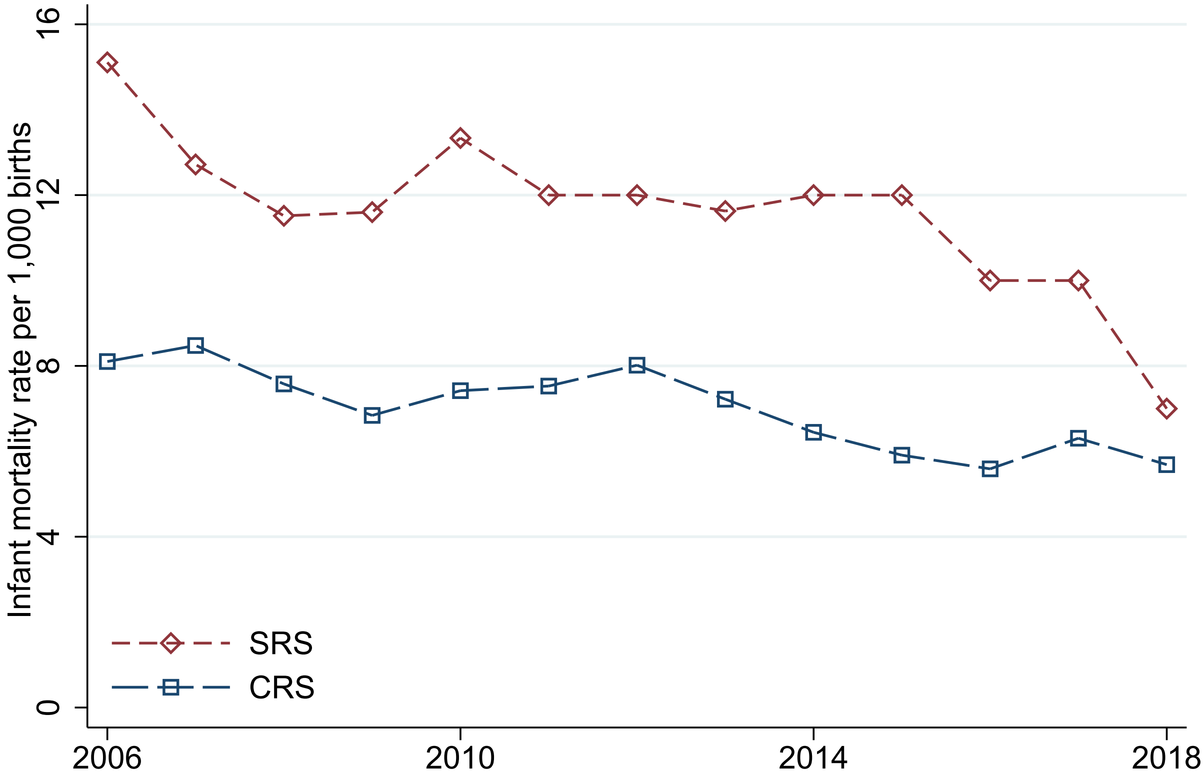

Civil Registration System (CRS) estimates are calculated by dividing infant deaths by births in a year.
Sample Registration System (SRS) rates are from annual summary reports prepared by the SRS.

Figure A5 Comparison of three-year average estimates of ${}_{n}m_{x}$ from civil and sample registration systems

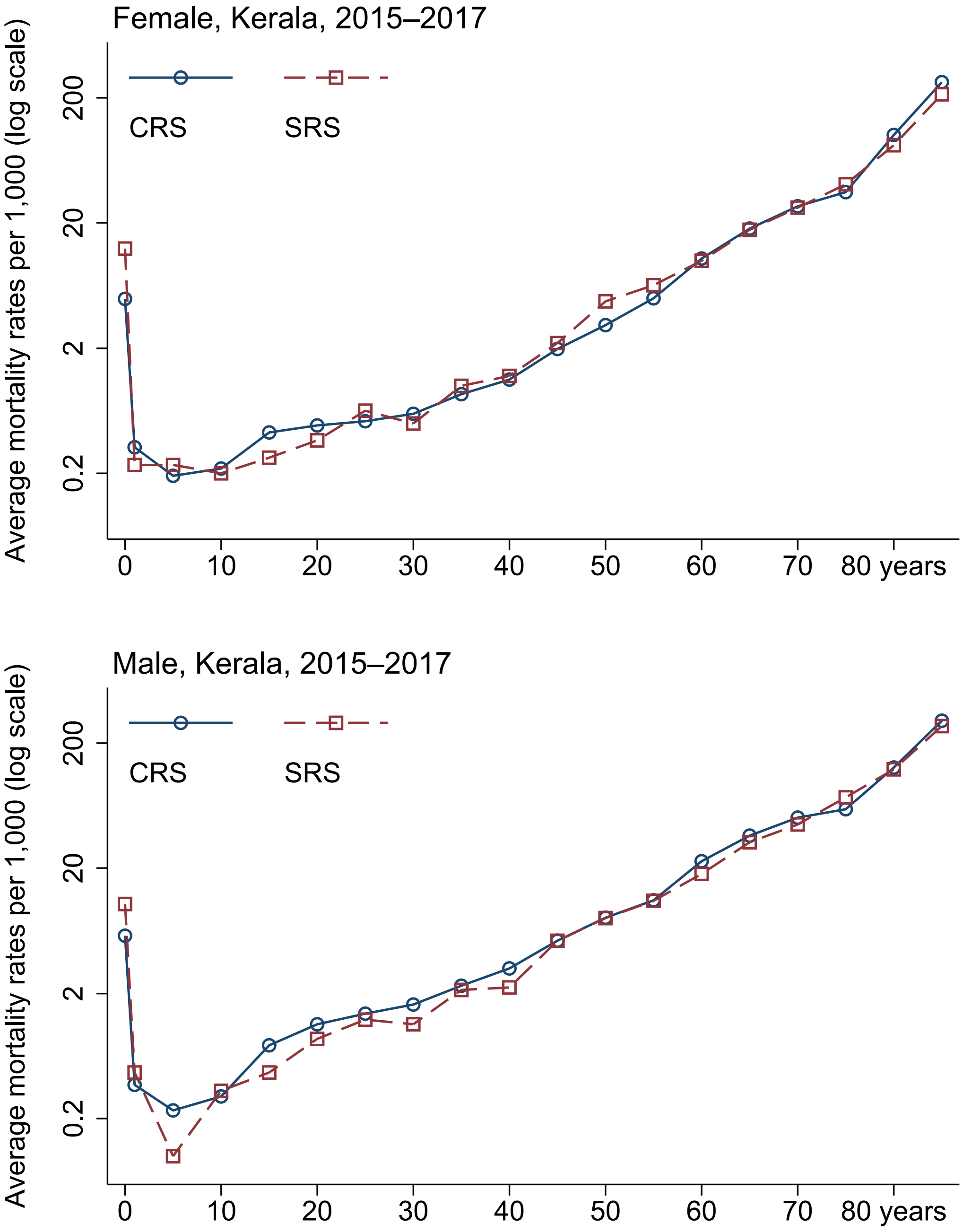

Sample Registration System (SRS) mortality rates are three-year averages from SRS statistical reports for 2015–2017. Civil Registration System (CRS) mortality rates are the three year average rates for 2015–2017.

Figure A6 Comparison of five-year average estimates of ${}_nm_x$ from civil and sample registration systems

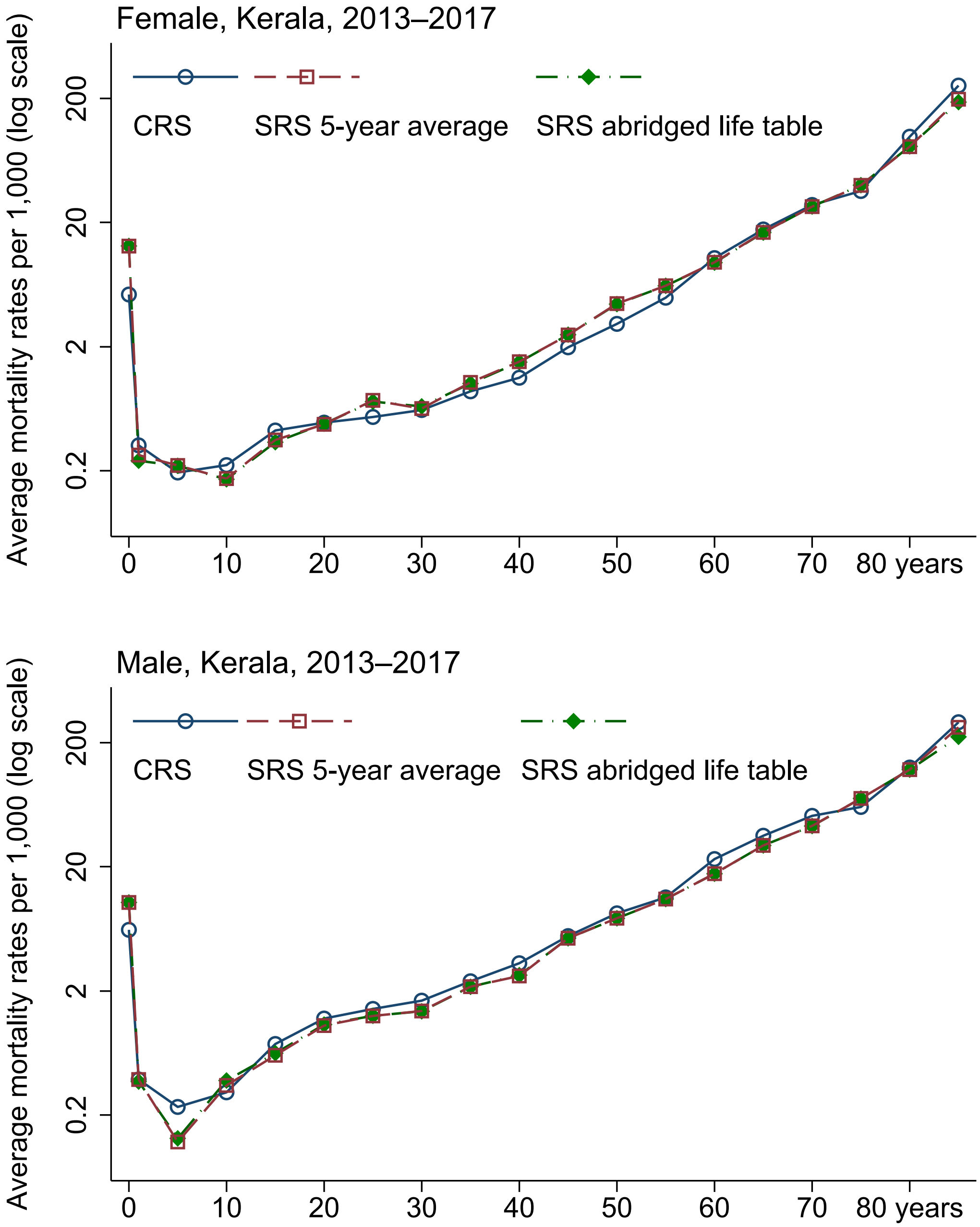

Sample Registration System (SRS) mortality rates are the five-year average rates calculated using annual SRS statistical reports for 2013–2017. Civil registration System (CRS) mortality rates are for the five-year period 2013–2017 and use population estimates calculated by the DHS Program (2020). The SRS abridged life table mortality rates are for 2013–2017.

Figure A7 Census vs estimated exposures (2011, mid year)

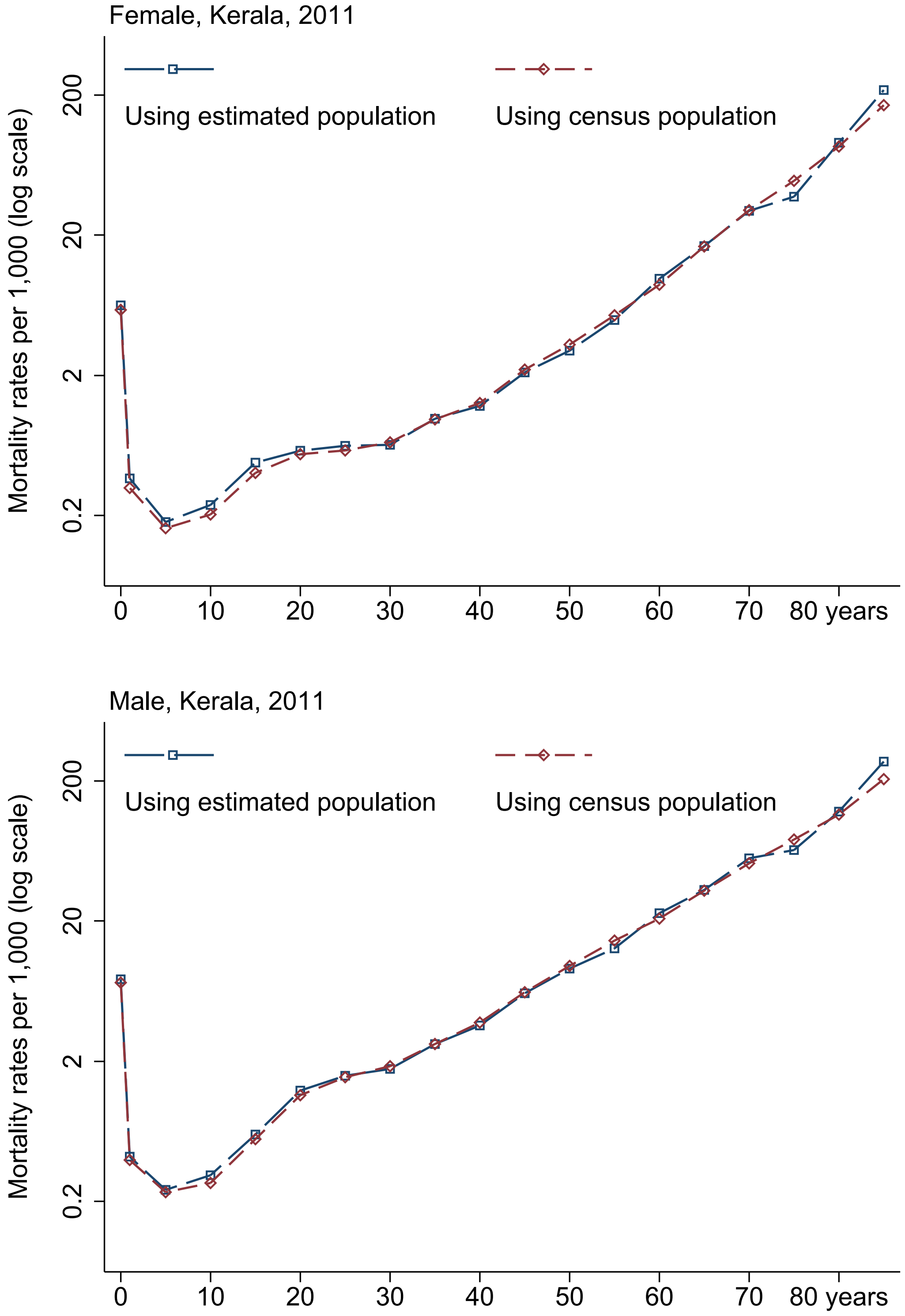

Estimated population rates are calculated by dividing deaths for the calendar year by the population estimated by the DHS Program (2020). Census population rates are calculated by dividing deaths from September 1, 2010 to August 31, 2011 by the 2011 census population.

Figure A8 Official projections vs estimated exposures (2016)

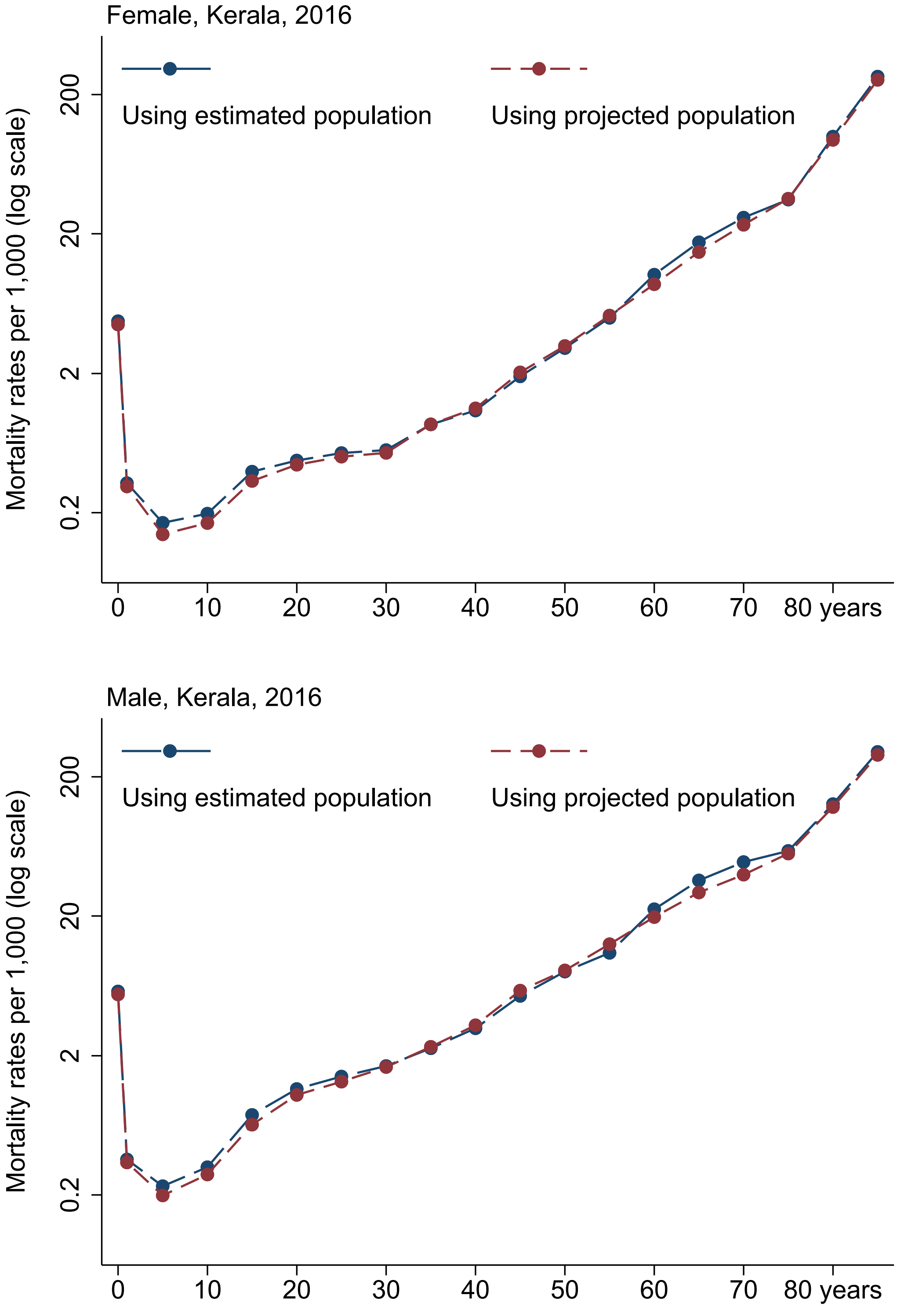

Estimated population rates are calculated by dividing deaths for the calendar year by the population estimated by the DHS Program (2020). Census population rates are calculated by dividing deaths for the calendar year by the projected census population for 2016.

Figure A9 Age distribution of deaths, female, 2006–2017

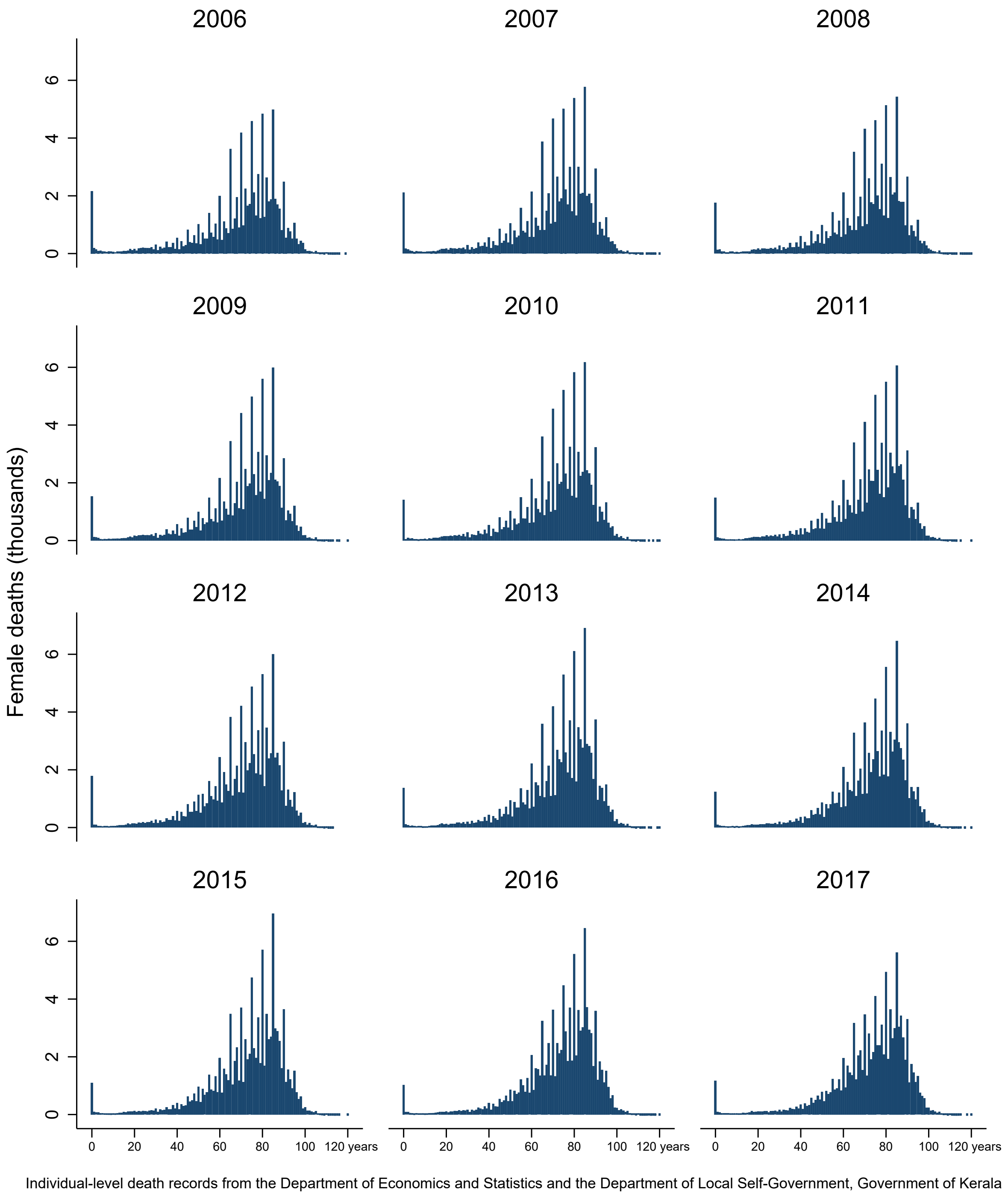

Figure A10 Age distribution of deaths, male, 2006–2017

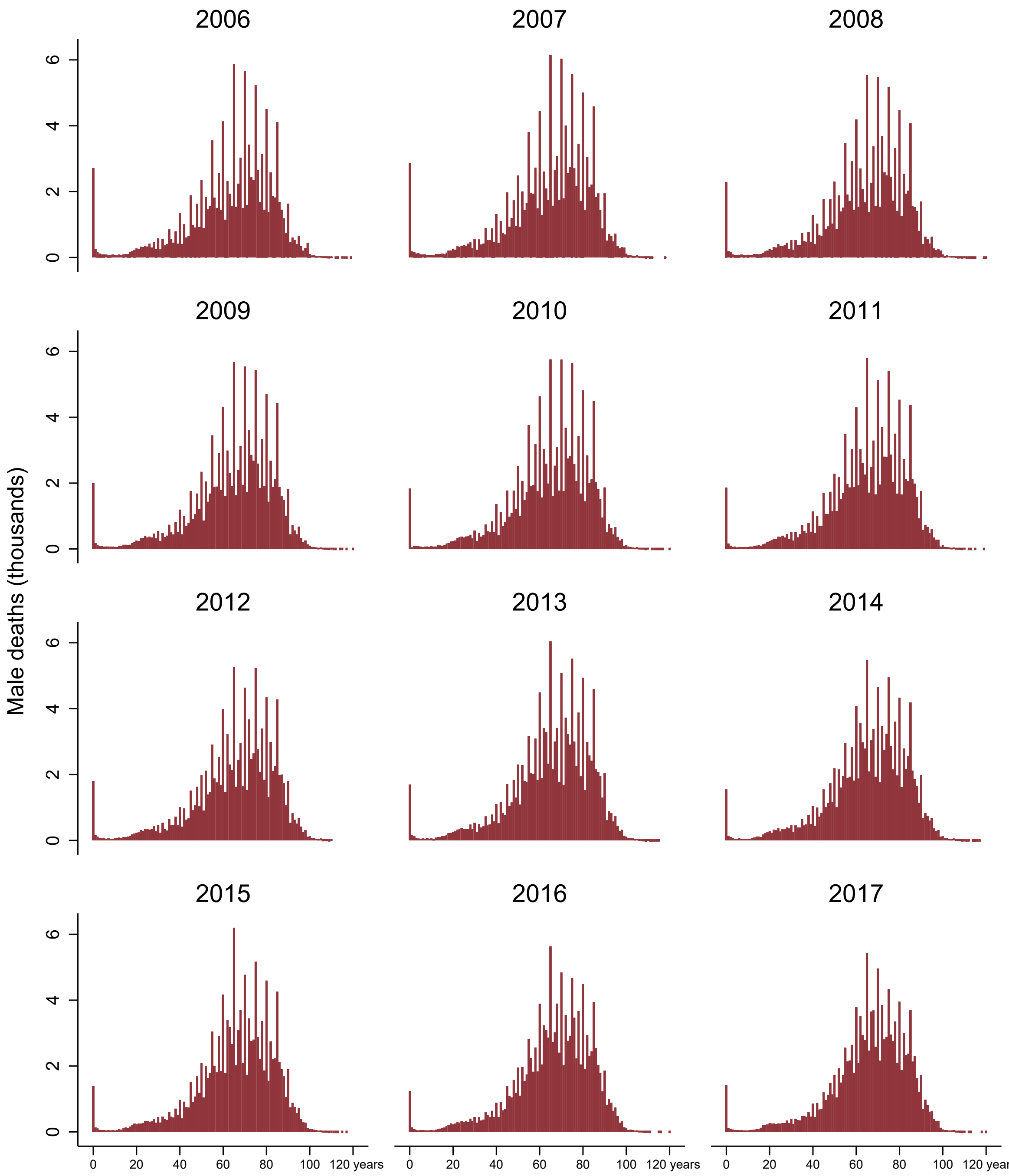

Individual-level death records from the Department of Economics and Statistics and the Department of Local Self-Government, Government of Kerala

Figure A11 Age heaping: Myers blended index and Whipple index

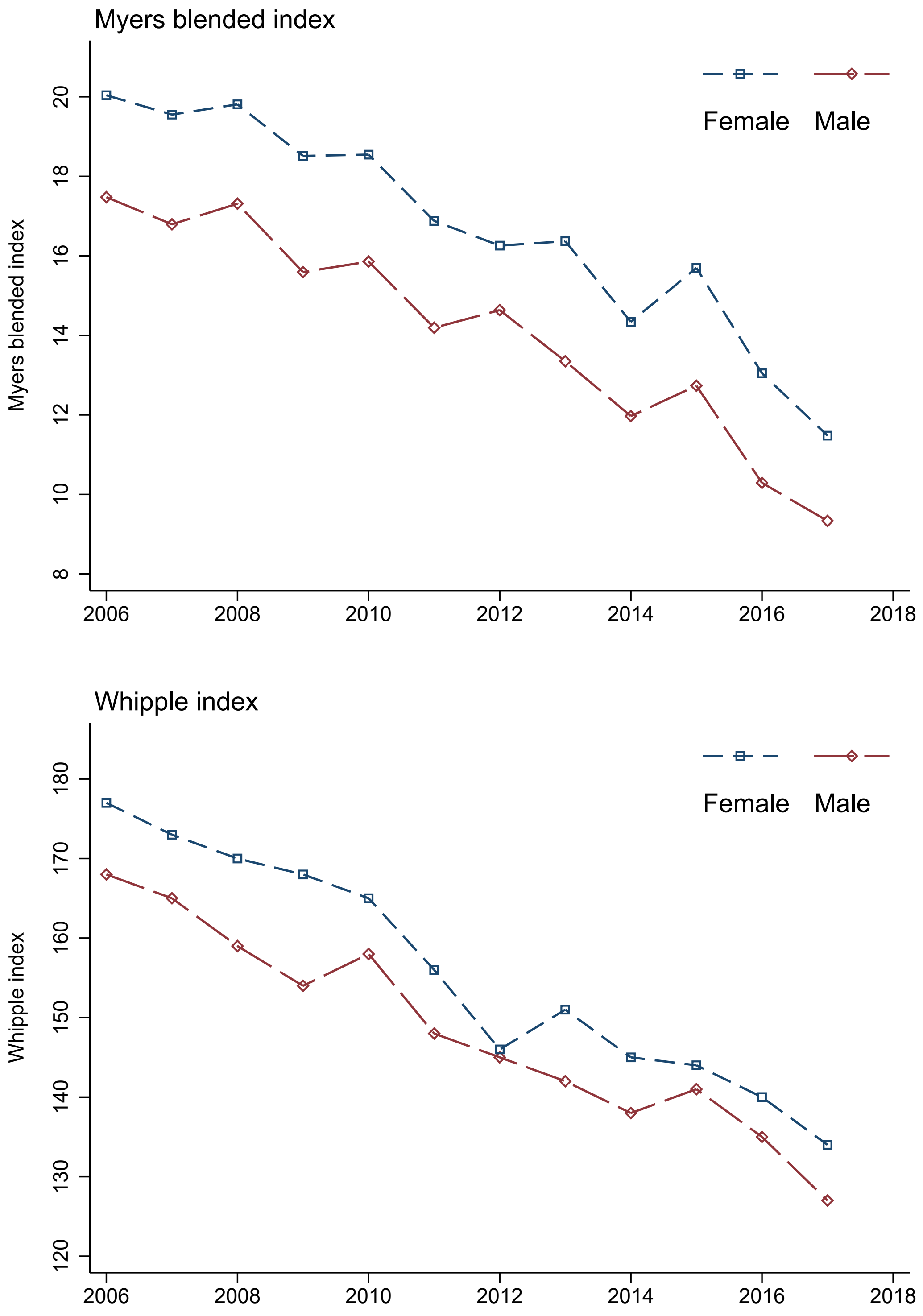

The Myers index considers preference of ages ending in each of the digits 0 to 9. The index ranges from 0 to 90. 0 indicates no age heaping and 90 indicates extreme age heaping. Whipple's index indicates the preference of ages ending in 0 or 5 for ages between 23 and 62. The index ranges from 100 to 500, with 100 representing no digit preference.

Figure A12 Comparison of unadjusted and Gompertz life-table estimates, single age years, 2010–2011

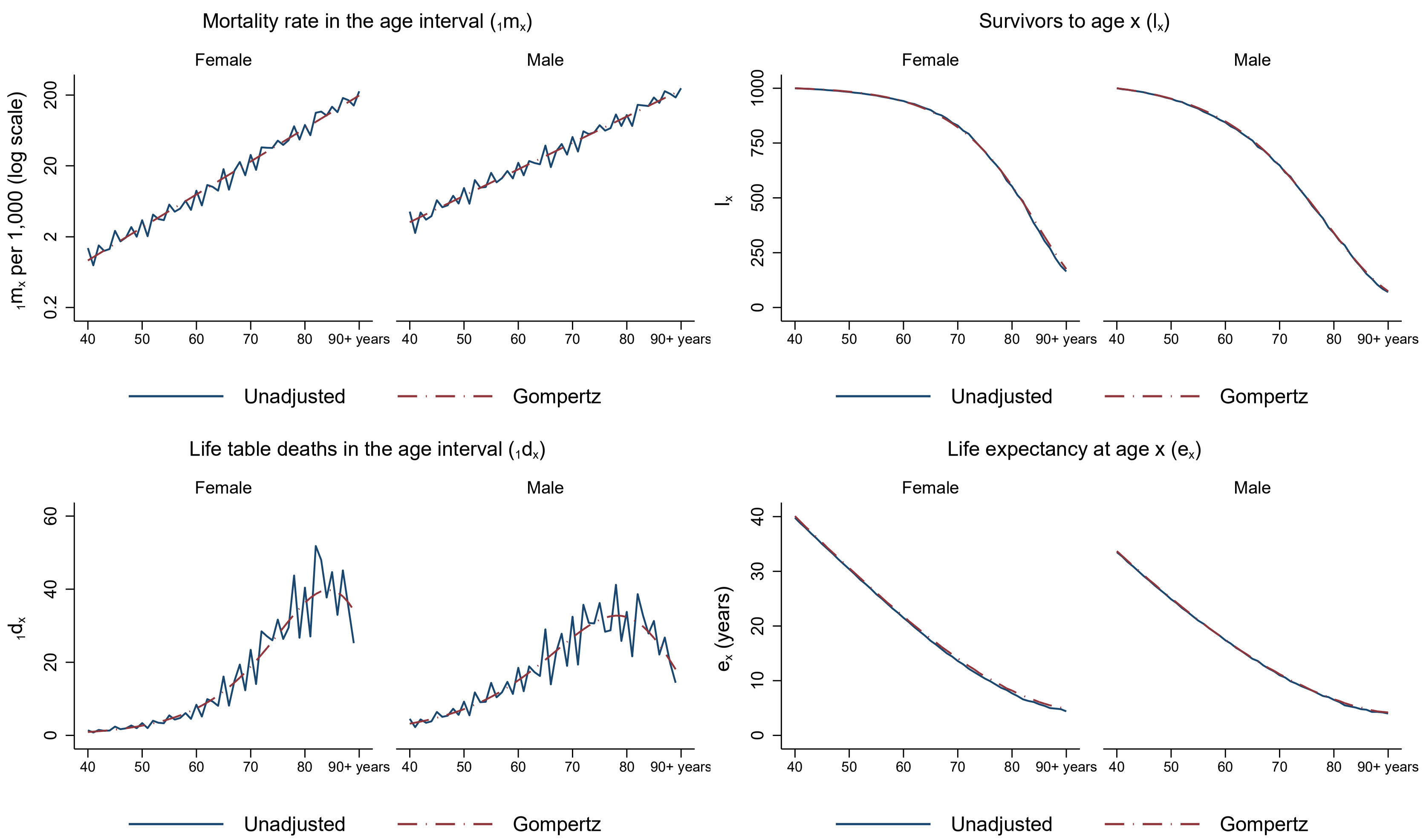

Note: Radix in the l_x graphs starts at age 40. The open-ended age interval is age 90. Age groups in x axis are in single age years. Unadusted rates use Census 2011 as exposures and deaths in the period 1 September 2010 to 31 August 2011. Gompertz mortality rates are predicted age-specific mortality rates using the unadjusted mortality rates in single ages. Both sets of life tables assume an $_na_x$ of 0.5.

Figure A13 Comparison of the relationship between child and adult mortality in Kerala and Human Mortality Database countries

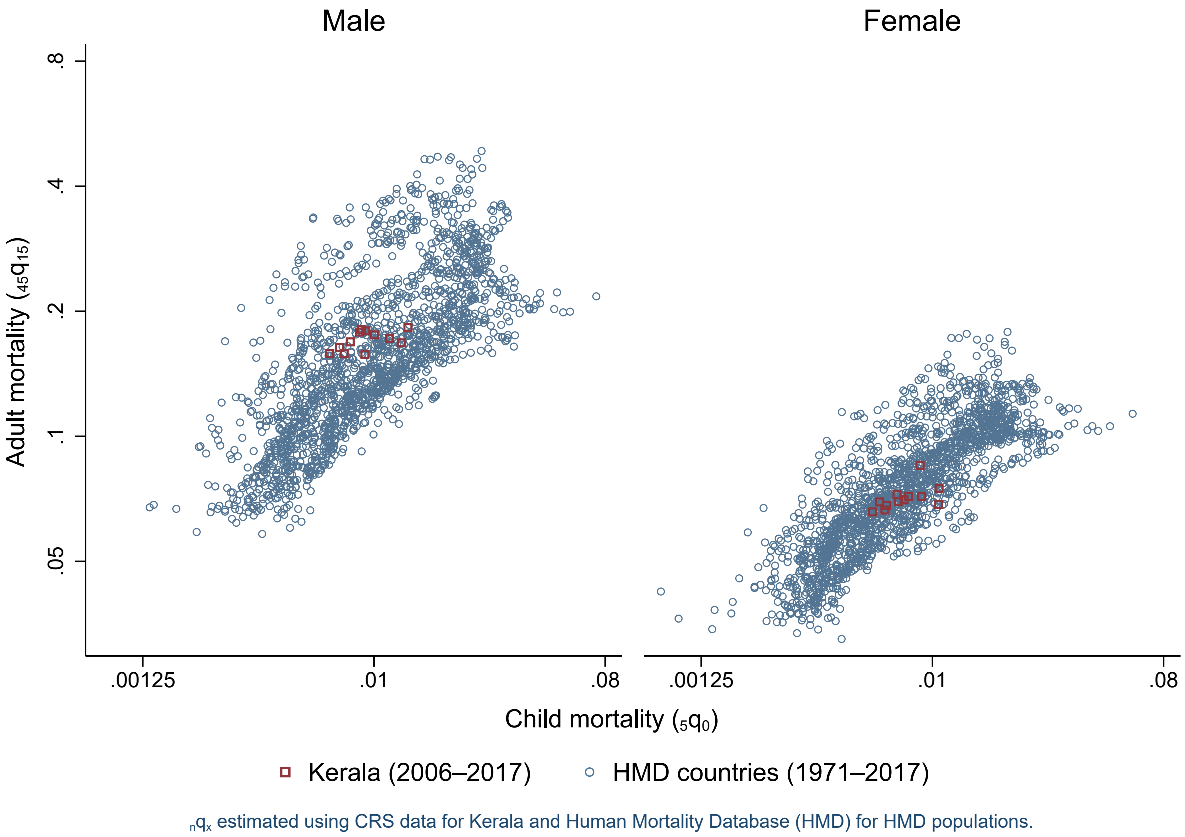

Figure A14 Comparison of the relationship between child and old-age mortality in Kerala and Human Mortality Database countries

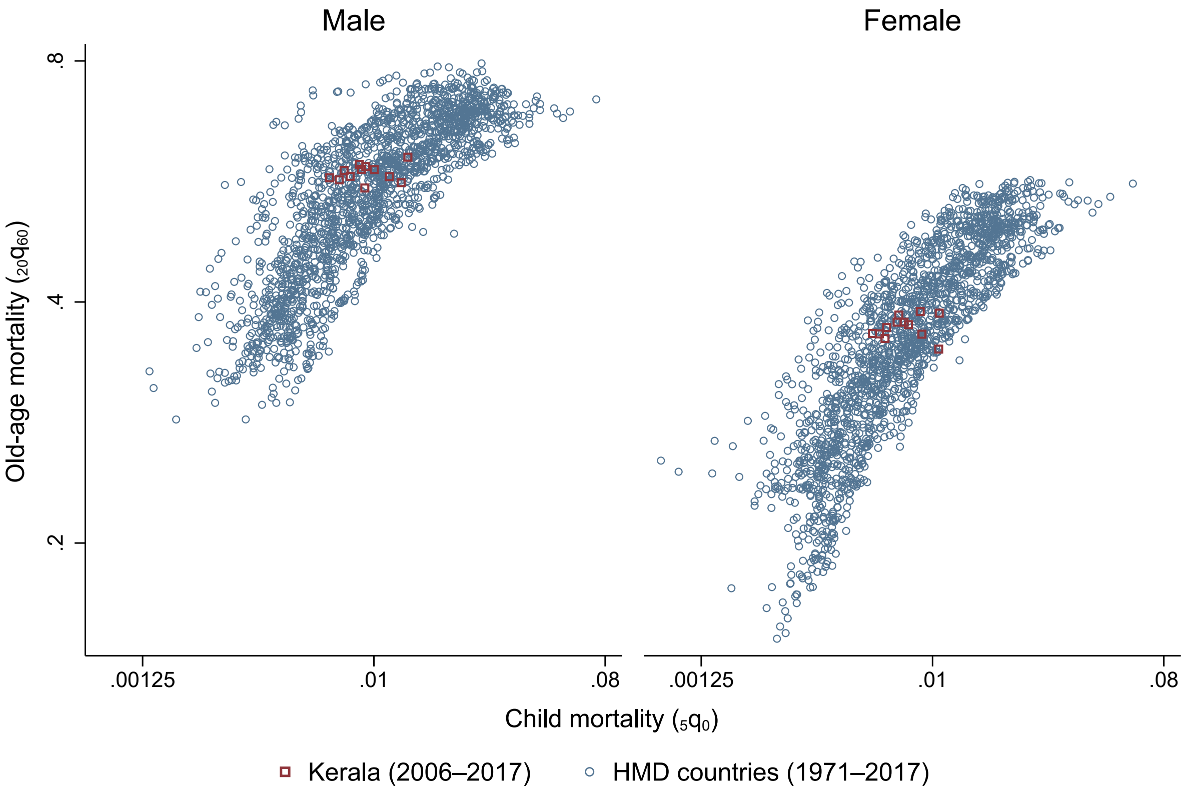

$_{n}q_{x}$ estimated using CRS data for Kerala and Human Mortality Database (HMD) for HMD populations.

Table A1 Observations with missing data, 2006–2017

Year	Only age (%)			Only sex	Sex and age	Sex or age
	Female	Male	Total	%	%	%
2006	0.03	0.02	0.02	1.97	0.00	1.99
2007	0.49	0.41	0.44	1.23	0.00	1.66
2008	0.53	0.52	0.52	0.61	0.00	1.13
2009	0.68	0.64	0.66	0.32	0.01	0.98
2010	0.86	0.84	0.85	0.06	0.02	0.91
2011	0.70	0.63	0.66	0.01	0.01	0.67
2012	0.57	0.52	0.54	0.01	0.00	0.55
2013	0.50	0.45	0.47	0.01	0.00	0.48
2014	0.45	0.39	0.41	0.01	0.00	0.42
2015	0.49	0.46	0.47	0.01	0.00	0.48
2016	0.49	0.44	0.46	0.00	0.00	0.46
2017	0.32	0.31	0.31	0.01	0.00	0.32

Note: Authors' calculations using data from the Kerala MARANAM Study and adjusted population estimates from the DHS Program (2020). Column 2 (3) refers to the share of female (male) deaths with missing age. Columns 4 to 7 refer to the share of total deaths with missing observations for the specified category.

Table A2 Life tables, 2011

	Female					Male				
Age x	$_nm_x$	$_nq_x$	l_x	$_nL_x$	e_x	$_nm_x$	$_nq_x$	l_x	$_nL_x$	e_x
0	0.0063	0.0063	1000	994.3	78.2	0.0077	0.0076	1000	992.9	70.9
1	0.0004	0.0015	994	3971.2	77.7	0.0004	0.0017	992	3965.5	70.5
5	0.0002	0.0009	992	4959.0	73.8	0.0002	0.0012	991	4950.5	66.6
10	0.0002	0.0012	991	4953.9	68.9	0.0003	0.0015	990	4943.7	61.7
15	0.0005	0.0024	990	4945.5	64.0	0.0006	0.0030	988	4933.5	56.7
20	0.0006	0.0029	988	4932.1	59.1	0.0012	0.0062	985	4911.0	51.9
25	0.0006	0.0031	985	4917.6	54.3	0.0016	0.0078	979	4876.0	47.2
30	0.0006	0.0032	982	4901.9	49.4	0.0018	0.0088	971	4836.2	42.6
35	0.0010	0.0049	979	4882.2	44.6	0.0027	0.0132	963	4784.1	37.9
40	0.0012	0.0060	974	4856.3	39.8	0.0036	0.0178	950	4710.5	33.4
45	0.0021	0.0104	968	4816.8	35.0	0.0061	0.0301	933	4599.6	28.9
50	0.0030	0.0149	958	4756.5	30.4	0.0092	0.0449	905	4429.2	24.8
55	0.0050	0.0245	944	4665.5	25.8	0.0128	0.0621	864	4196.1	20.8
60	0.0098	0.0479	921	4501.7	21.4	0.0228	0.1082	811	3846.2	17.0
65	0.0167	0.0806	876	4220.4	17.3	0.0334	0.1549	723	3349.3	13.7
70	0.0299	0.1397	806	3766.8	13.6	0.0562	0.2477	611	2691.7	10.8
75	0.0377	0.1732	693	3182.4	10.3	0.0644	0.2776	460	1981.9	8.5
80	0.0915	0.3740	573	2341.8	7.0	0.1212	0.4625	332	1267.4	5.8
85	0.2178	1.0000	359	1647.5	4.6	0.2758	1.0000	178	647.2	3.6

Note: Authors' calculations using data from the Kerala MARANAM Study and adjusted population estimates from DHS Program (2020).

Table A3 Life tables, 2012

	Female					Male				
Age x	${}_nm_x$	${}_nq_x$	l_x	${}_nL_x$	e_x	${}_nm_x$	${}_nq_x$	l_x	${}_nL_x$	e_x
0	0.0077	0.0077	1000	993.0	77.5	0.0076	0.0076	1000	993.0	72.3
1	0.0003	0.0013	992	3966.1	77.1	0.0004	0.0017	992	3965.8	71.8
5	0.0002	0.0010	991	4952.7	73.2	0.0002	0.0011	991	4951.1	67.9
10	0.0003	0.0014	990	4946.6	68.3	0.0003	0.0016	990	4944.3	63.0
15	0.0005	0.0024	989	4937.7	63.3	0.0006	0.0030	988	4933.8	58.1
20	0.0006	0.0032	986	4924.0	58.5	0.0011	0.0057	985	4912.3	53.3
25	0.0007	0.0035	983	4907.7	53.7	0.0014	0.0072	979	4880.1	48.6
30	0.0008	0.0040	980	4889.3	48.8	0.0016	0.0078	972	4843.9	43.9
35	0.0011	0.0054	976	4866.6	44.0	0.0023	0.0113	965	4798.3	39.2
40	0.0015	0.0075	971	4836.1	39.3	0.0032	0.0157	954	4734.2	34.6
45	0.0026	0.0129	963	4787.7	34.5	0.0053	0.0263	939	4636.4	30.2
50	0.0038	0.0189	951	4712.5	29.9	0.0079	0.0387	914	4488.1	25.9
55	0.0062	0.0306	933	4599.2	25.5	0.0108	0.0527	879	4286.0	21.8
60	0.0119	0.0579	904	4402.0	21.2	0.0204	0.0973	833	3971.0	17.9
65	0.0184	0.0883	852	4087.5	17.3	0.0300	0.1400	752	3507.5	14.5
70	0.0303	0.1416	777	3627.6	13.7	0.0491	0.2197	646	2891.2	11.5
75	0.0373	0.1715	667	3063.8	10.6	0.0612	0.2659	504	2192.4	9.0
80	0.0883	0.3632	553	2272.1	7.2	0.1177	0.4528	370	1424.4	6.3
85	0.2056	1.0000	352	1711.1	4.9	0.2216	1.0000	203	914.3	4.5

Note: Authors' calculations using data from the Kerala MARANAM Study and adjusted population estimates from the DHS Program (2020).

Table A4 Life tables, 2013

	Female					Male				
Age x	$_nm_x$	$_nq_x$	l_x	$_nL_x$	e_x	$_nm_x$	$_nq_x$	l_x	$_nL_x$	e_x
0	0.0061	0.0060	1000	994.5	78.0	0.0071	0.0071	1000	993.4	70.8
1	0.0003	0.0014	994	3972.5	77.5	0.0004	0.0017	993	3967.7	70.3
5	0.0002	0.0010	993	4960.5	73.6	0.0002	0.0012	991	4953.1	66.5
10	0.0002	0.0012	992	4955.1	68.7	0.0003	0.0015	990	4946.2	61.5
15	0.0004	0.0022	990	4947.3	63.8	0.0007	0.0035	988	4934.6	56.6
20	0.0005	0.0025	988	4935.5	58.9	0.0013	0.0065	985	4909.8	51.8
25	0.0006	0.0031	986	4922.0	54.0	0.0016	0.0078	979	4874.4	47.1
30	0.0007	0.0034	983	4906.2	49.2	0.0018	0.0089	971	4834.7	42.5
35	0.0009	0.0047	980	4886.5	44.3	0.0026	0.0129	962	4782.8	37.8
40	0.0011	0.0057	975	4861.6	39.5	0.0038	0.0186	950	4708.4	33.3
45	0.0020	0.0100	969	4824.5	34.8	0.0061	0.0302	932	4595.9	28.9
50	0.0031	0.0155	960	4763.5	30.1	0.0092	0.0450	904	4425.7	24.7
55	0.0050	0.0246	945	4669.4	25.5	0.0122	0.0593	863	4197.8	20.7
60	0.0105	0.0512	921	4497.8	21.1	0.0239	0.1129	812	3844.6	16.9
65	0.0173	0.0831	874	4203.6	17.1	0.0349	0.1612	721	3325.5	13.7
70	0.0300	0.1403	802	3744.9	13.4	0.0541	0.2396	604	2674.9	10.8
75	0.0389	0.1780	689	3156.2	10.1	0.0655	0.2825	460	1981.0	8.4
80	0.0938	0.3819	566	2305.5	6.8	0.1261	0.4777	330	1249.2	5.7
85	0.2298	1.0000	350	1523.3	4.4	0.2732	1.0000	172	630.4	3.7

Note: Authors' calculations using data from the Kerala MARANAM Study and adjusted population estimates from the DHS Program (2020).

Table A5 Life tables, 2014

	Female					Male				
Age x	${}_nm_x$	${}_nq_x$	l_x	${}_nL_x$	e_x	${}_nm_x$	${}_nq_x$	l_x	${}_nL_x$	e_x
0	0.0054	0.0054	1000	995.1	78.4	0.0066	0.0066	1000	993.9	71.4
1	0.0003	0.0012	995	3975.6	77.9	0.0004	0.0015	993	3970.2	70.8
5	0.0002	0.0009	993	4965.0	73.9	0.0002	0.0011	992	4956.9	66.9
10	0.0002	0.0011	993	4959.9	69.0	0.0003	0.0016	991	4950.3	62.0
15	0.0004	0.0020	991	4952.5	64.1	0.0007	0.0036	989	4938.4	57.1
20	0.0005	0.0025	989	4941.4	59.2	0.0013	0.0064	986	4913.9	52.3
25	0.0005	0.0026	987	4928.7	54.4	0.0015	0.0073	979	4879.8	47.6
30	0.0006	0.0031	984	4914.4	49.5	0.0017	0.0083	972	4842.0	42.9
35	0.0009	0.0042	981	4896.8	44.6	0.0025	0.0122	964	4792.8	38.3
40	0.0011	0.0055	977	4873.3	39.8	0.0035	0.0172	952	4723.9	33.7
45	0.0020	0.0098	972	4837.1	35.0	0.0057	0.0283	936	4619.3	29.3
50	0.0030	0.0148	962	4778.4	30.3	0.0089	0.0435	910	4454.8	25.0
55	0.0048	0.0238	948	4687.6	25.8	0.0113	0.0549	870	4238.6	21.1
60	0.0100	0.0490	926	4523.1	21.3	0.0234	0.1111	822	3897.1	17.1
65	0.0166	0.0798	880	4239.1	17.3	0.0336	0.1558	731	3383.5	13.9
70	0.0272	0.1276	810	3805.1	13.5	0.0511	0.2277	617	2748.2	11.0
75	0.0351	0.1620	707	3262.9	10.1	0.0608	0.2649	477	2076.5	8.5
80	0.0958	0.3879	592	2398.8	6.6	0.1226	0.4677	350	1336.4	5.7
85	0.2413	1.0000	362	1502.4	4.1	0.2886	1.0000	186	646.1	3.5

Note: Authors' calculations using data from the Kerala MARANAM Study and adjusted population estimates from the DHS Program (2020).

Table A6 Life tables, 2015

	Female					Male				
Age x	${}_nm_x$	${}_nq_x$	l_x	${}_nL_x$	e_x	${}_nm_x$	${}_nq_x$	l_x	${}_nL_x$	e_x
0	0.0049	0.0049	1000	995.5	78.2	0.0059	0.0058	1000	994.6	71.6
1	0.0003	0.0013	995	3977.2	77.5	0.0004	0.0015	994	3973.1	71.0
5	0.0002	0.0008	994	4966.9	73.6	0.0002	0.0011	993	4960.6	67.1
10	0.0002	0.0011	993	4962.0	68.7	0.0003	0.0015	992	4954.2	62.2
15	0.0004	0.0022	992	4954.1	63.8	0.0007	0.0037	990	4942.1	57.3
20	0.0005	0.0025	990	4942.3	58.9	0.0011	0.0055	986	4919.4	52.5
25	0.0005	0.0026	987	4929.4	54.0	0.0014	0.0070	981	4888.1	47.8
30	0.0006	0.0031	985	4915.3	49.2	0.0017	0.0084	974	4850.9	43.1
35	0.0009	0.0044	981	4897.4	44.3	0.0024	0.0118	966	4802.7	38.4
40	0.0011	0.0056	977	4873.2	39.5	0.0033	0.0162	955	4736.9	33.9
45	0.0020	0.0101	972	4836.0	34.7	0.0054	0.0268	939	4637.7	29.4
50	0.0032	0.0157	962	4774.2	30.1	0.0084	0.0410	914	4481.0	25.1
55	0.0051	0.0252	947	4677.6	25.5	0.0113	0.0552	876	4268.6	21.1
60	0.0100	0.0490	923	4509.8	21.1	0.0229	0.1085	828	3931.1	17.2
65	0.0180	0.0863	878	4212.8	17.0	0.0357	0.1645	738	3402.4	13.9
70	0.0271	0.1272	802	3767.6	13.4	0.0492	0.2200	617	2757.4	11.1
75	0.0352	0.1627	700	3231.9	9.9	0.0588	0.2574	481	2104.6	8.6
80	0.0986	0.3975	586	2362.2	6.4	0.1235	0.4702	357	1360.6	5.6
85	0.2583	1.0000	353	1367.2	3.9	0.2909	1.0000	189	650.8	3.4

Note: Authors' calculations using data from the Kerala MARANAM Study and adjusted population estimates from the DHS Program (2020).

Table A7 Life tables, 2016

	Female					Male				
Age x	${}_nm_x$	${}_nq_x$	l_x	${}_nL_x$	e_x	${}_nm_x$	${}_nq_x$	l_x	${}_nL_x$	e_x
0	0.0045	0.0045	1000	995.9	78.2	0.0053	0.0052	1000	995.1	71.7
1	0.0003	0.0013	995	3978.7	77.6	0.0004	0.0015	995	3975.5	71.1
5	0.0002	0.0009	994	4968.7	73.7	0.0002	0.0012	993	4963.5	67.2
10	0.0002	0.0010	993	4964.0	68.7	0.0003	0.0015	992	4956.8	62.3
15	0.0004	0.0019	992	4957.2	63.8	0.0008	0.0038	991	4944.3	57.4
20	0.0004	0.0022	990	4946.7	58.9	0.0012	0.0058	987	4920.6	52.6
25	0.0005	0.0027	988	4934.3	54.0	0.0014	0.0070	981	4888.6	47.9
30	0.0006	0.0029	985	4920.5	49.2	0.0016	0.0081	974	4852.3	43.2
35	0.0008	0.0041	983	4903.9	44.3	0.0023	0.0114	966	4805.9	38.5
40	0.0011	0.0054	979	4880.7	39.5	0.0031	0.0152	955	4743.0	33.9
45	0.0019	0.0094	973	4845.8	34.7	0.0053	0.0261	941	4648.0	29.4
50	0.0030	0.0148	964	4787.7	30.0	0.0080	0.0391	916	4496.3	25.1
55	0.0049	0.0240	950	4695.3	25.4	0.0108	0.0528	880	4292.1	21.0
60	0.0104	0.0509	927	4525.6	20.9	0.0224	0.1067	834	3963.7	17.1
65	0.0177	0.0848	880	4225.4	16.9	0.0361	0.1661	745	3431.0	13.8
70	0.0269	0.1263	805	3784.6	13.3	0.0499	0.2227	621	2771.8	11.0
75	0.0355	0.1638	704	3248.3	9.8	0.0597	0.2605	483	2108.0	8.4
80	0.1018	0.4081	588	2358.2	6.2	0.1281	0.4834	357	1347.6	5.5
85	0.2723	1.0000	348	1279.1	3.7	0.3068	1.0000	184	601.1	3.3

Note: Authors' calculations using data from the Kerala MARANAM Study and adjusted population estimates from the DHS Program (2020).

Table A8 Life tables, 2017

	Female					Male				
Age x	${}_nm_x$	${}_nq_x$	l_x	${}_nL_x$	e_x	${}_nm_x$	${}_nq_x$	l_x	${}_nL_x$	e_x
0	0.0054	0.0054	1000	995.1	77.9	0.0062	0.0062	1000	994.3	71.4
1	0.0003	0.0013	995	3975.5	77.3	0.0004	0.0015	994	3971.8	70.9
5	0.0002	0.0011	993	4964.1	73.4	0.0002	0.0012	992	4958.9	67.0
10	0.0002	0.0011	992	4958.5	68.5	0.0003	0.0015	991	4952.1	62.1
15	0.0004	0.0022	991	4951.0	63.6	0.0008	0.0040	990	4939.0	57.2
20	0.0005	0.0025	989	4939.0	58.7	0.0011	0.0056	986	4915.2	52.4
25	0.0005	0.0024	986	4926.4	53.8	0.0013	0.0065	980	4884.8	47.7
30	0.0006	0.0030	984	4913.4	49.0	0.0016	0.0079	974	4850.3	43.0
35	0.0009	0.0043	981	4896.1	44.1	0.0023	0.0112	966	4805.2	38.3
40	0.0012	0.0058	977	4871.4	39.3	0.0032	0.0158	955	4741.8	33.7
45	0.0020	0.0099	971	4834.6	34.5	0.0051	0.0254	940	4647.1	29.2
50	0.0030	0.0150	962	4774.6	29.8	0.0078	0.0385	916	4498.1	24.9
55	0.0051	0.0250	947	4679.5	25.2	0.0111	0.0542	881	4292.2	20.8
60	0.0108	0.0525	924	4504.6	20.8	0.0230	0.1094	833	3957.4	16.8
65	0.0186	0.0889	875	4193.1	16.8	0.0377	0.1731	742	3406.6	13.5
70	0.0278	0.1303	797	3739.1	13.2	0.0533	0.2362	614	2717.4	10.8
75	0.0353	0.1630	693	3203.7	9.8	0.0596	0.2601	469	2046.4	8.4
80	0.1030	0.4122	580	2322.3	6.2	0.1316	0.4933	347	1300.2	5.4
85	0.2724	1.0000	341	1252.1	3.7	0.3090	1.0000	176	568.6	3.2

Note: Authors' calculations using data from the Kerala MARANAM Study and adjusted population estimates from the DHS Program (2020).

Table A9 Crude death rate estimates adjusted for migration (2011); by sex

	Female	Male	Total
Crude death rate (CDR)[a]	5.93	8.64	7.23
CDR excluding migrants[b]	5.99	8.70	7.29
CDR excluding recent migrants[c]	5.89	8.54	7.16

[a] Census populations, all civil registration system deaths.
[b] Deaths are for people with permanent residence in Kerala, and population counts exclude people born outside Kerala.
[c] Deaths are for people with permanent residence in Kerala, and population counts exclude people whose last residence was outside Kerala in the last year.

Table A10 Standard deviation, mortality rate (${}_nm_x$), 2006–2017

	CRS		SRS	
Age x	Female	Male	Female	Male
0	1.38	1.88	1.13	1.09
1	0.08	0.08	0.21	0.19
5	0.04	0.04	0.09	0.11
10	0.03	0.02	0.11	0.12
15	0.04	0.07	0.18	0.27
20	0.08	0.06	0.28	0.32
25	0.07	0.08	0.30	0.43
30	0.07	0.07	0.26	0.45
35	0.09	0.17	0.25	0.42
40	0.12	0.29	0.50	0.77
45	0.18	0.40	0.55	0.88
50	0.24	0.51	1.19	0.96
55	0.40	0.82	0.79	1.20
60	0.98	1.76	1.44	1.81
65	0.80	2.15	1.73	2.63
70	1.59	3.45	4.91	6.66
75	1.61	2.97	8.81	6.50
80	7.64	8.10	11.68	13.54
85	31.51	31.92	29.63	36.72

Note: Authors' calculations using data from the Kerala MARANAM Study and adjusted population estimates from the DHS Program (2020). CRS: Civil Registration System. SRS: Sample Registration System.
